# Ischemia–Reperfusion Injury: Molecular Mechanisms and Therapeutic Interventions

**DOI:** 10.1002/mco2.70822

**Published:** 2026-06-17

**Authors:** Peng An, Yi An, Mengwei Chen, Longlong Wu, Rong Wang

**Affiliations:** ^1^ The Gastroenterology Department of Shanxi Provincial People's Hospital Shanxi Medical University Taiyuan China; ^2^ Taiyuan Peace Hospital Taiyuan China

**Keywords:** ischemia–reperfusion injury, immunothrombosis, mitochondrial dysfunction, neutrophil extracellular traps, organ transplantation, regulated cell death

## Abstract

Ischemia–reperfusion injury (IRI) is a common pathological process underlying cardiovascular events, organ transplantation, and shock resuscitation. Its paradox is that restoration of blood flow, while essential for tissue survival, can itself intensify cellular stress and amplify tissue damage. This review delineates the cross‐organ mechanistic cascade of IRI, beginning with metabolic collapse, adenosine triphosphate (ATP) depletion, and ionic dysregulation during ischemia, and progressing to reperfusion‐driven mitochondrial dysfunction, oxidative and nitrosative stress, and activation of regulated cell death programs. We further highlight how sterile inflammation evolves into a coordinated endothelial–immune–coagulation interaction network centered on neutrophil extracellular traps (NETs), which couple microvascular obstruction with inflammatory signaling to promote immunothrombosis and propagate injury locally and to distant organs. Despite major advances in mechanistic insight, translation into effective therapies remains inconsistent, largely due to phenotypic heterogeneity, narrow therapeutic windows, and the lack of real‐time biomarkers that capture pathway activity. Future progress will require a systems‐level, cross‐organ framework for immune remodeling, with particular emphasis on targetable NET‐driven immunothrombotic phenotypes. Biomarker‐guided precision stratification, multimodal data integration, ex vivo machine perfusion platforms, and organoid‐based human models may enable mechanism‐aligned trials and shift therapy from macroscopic reperfusion toward microvascular stabilization and cellular repair.

## Graphical abstract

Multiorgan ischemia–reperfusion injury begins with ischemia‐induced ATP depletion and ionic imbalance, followed by reperfusion‐triggered mitochondrial ROS/RNS bursts, regulated cell death, and DAMP release. Sterile inflammation converges on endothelial–immune–coagulation crosstalk, where NETs drive immunothrombosis, no‐reflow, and remote organ injury. Biomarker‐defined endotypes may guide timed reperfusion modulation, mitochondrial protection, complement blockade, and NET‐targeted therapy.

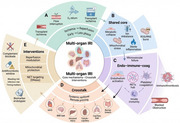

## 1 Introduction

This introduction frames ischemia–reperfusion injury (IRI) as a cross‐organ reperfusion paradox and explains why a unified mechanistic and translational perspective is needed. It highlights the recurring metabolic, inflammatory, and immunothrombotic axes that structure the review.

IRI embodies a fundamental paradox in modern medicine's principle of “restoring blood flow to salvage tissue”: although reperfusion is essential for tissue survival, it frequently initiates a cascade of complex pathophysiological reactions that exacerbate tissue injury and, in severe cases, precipitate multiple organ dysfunction syndrome (MODS). From thrombolytic or interventional reperfusion in myocardial infarction and mechanical thrombectomy in ischemic stroke to liver and kidney transplantation, shock resuscitation, and cardiopulmonary bypass, IRI pervades a wide spectrum of clinical contexts, shaping disease progression and therapeutic outcomes [1, 2]. Importantly, the impact of IRI extends beyond the initially ischemic organ. Although reperfusion injury shares conserved cross‐organ mechanisms, it manifests distinct pathological phenotypes owing to differences in organ architecture, cellular composition, and metabolic demands, thereby posing substantial challenges for both basic investigation and clinical translation [1].

Over several decades, research has reshaped the conceptual framework of IRI from the classical notion that “ischemia itself causes injury” to a paradigm emphasizing that reperfusion induces marked metabolic and immune reprogramming. At the molecular level, multiomics analyses have identified a conserved metabolic signature during ischemia across multiple tissues, characterized by marked succinate accumulation during the ischemic phase and its rapid mitochondrial oxidation upon reperfusion [3]. This process drives a burst of reactive oxygen species (ROS) via reverse electron transport (RET) at mitochondrial Complex I, providing a unifying biochemical explanation for the exacerbation of tissue injury upon reperfusion in organs such as the heart, brain, kidney, and liver [4]. These findings underscore the cross‐organ consistency of metabolically triggered IRI [3, 4]. In parallel, advances in immunology have revealed that sterile inflammation extends beyond the local release of danger signals from injured cells to form an intercellular—and even systemic—“danger signal network.” Damage‐associated molecular patterns (DAMPs) released from ischemic tissues disseminate through the circulation or lymphatic system, provoking systemic inflammatory responses and injury in distant organs [5, 6]. For example, neutrophil activation and complement cascade engagement following localized ischemia–reperfusion can promote microvascular endothelial activation and immune cell recruitment in remote tissues, ultimately leading to secondary organ dysfunction [7]. Collectively, these insights establish IRI not as an isolated, organ‐confined insult, but as a modular and reusable pathological process whose manifestations are shaped by tissue‐specific architecture and immune context [5].

Within this shared pathophysiological framework, neutrophil extracellular traps (NETs) and NET‐mediated immunothrombosis have attracted increasing attention in recent years, emerging as a major amplifying axis underlying the cross‐organ consistency of IRI [8, 9]. NETs are web‐like chromatin structures released by activated neutrophils during sterile inflammation and are decorated with histones and granular proteases, thereby providing a structural scaffold for platelet adhesion and coagulation factor deposition [9]. Excessive NET accumulation can directly obstruct the microvascular lumen and injure endothelial cells, leading to impaired perfusion and tissue hypoxia. In parallel, NET‐associated proteins exert potent cytotoxic and proinflammatory effects, thereby sustaining and amplifying inflammatory responses. Through these mechanisms, NETs function as a mechanistic link between innate immunity and the coagulation cascade, converting localized sterile inflammation into systemic immune–coagulation dysregulation [10]. Contrary to the traditional view that inflammation and coagulation proceed as parallel but independent processes, accumulating evidence indicates that they form a tightly coupled positive feedback network during IRI [10]. Activated endothelial cells provide a prothrombotic surface that facilitates leukocyte adhesion and coagulation activation, while the complement–platelet–coagulation factor axis further accelerates thrombus formation. Within this network, NETs provide a scaffold that integrates these pathways into a self‐sustaining pathological circuit. This immunothrombotic network underlies the “no‐reflow” phenomenon—where macrovascular patency is restored but microvascular perfusion remains compromised—highlighting microcirculatory dysfunction as a major bottleneck in IRI. The NET‐driven immunothrombotic paradigm also explains why therapies targeting inflammation or coagulation alone often fail to achieve clinical efficacy, as IRI fundamentally arises from a synergistic imbalance within the vascular–immune–coagulation triad rather than dysfunction of a single pathway. Consequently, increasing attention has been directed toward the kinetics of NET formation, their organ‐specific modifications, and their interactions with tissue‐resident immune cells, as these features may elucidate the heterogeneous origins of IRI across organs and reveal novel targets for precision intervention.

Despite rapid progress in mechanistic research, the field continues to face a major translational challenge, often described as the dilemma of “strong mechanisms but weak translation.” Biological heterogeneity among patients, organs, and reperfusion strategies has contributed to the frequent failure of single‐target interventions that demonstrate efficacy in animal models to reproduce comparable benefits in clinical trials. Conversely, conventional clinical endpoints—such as isolated organ function indices or short‐term survival—often fail to capture critical intermediate phenotypes, including microcirculatory impairment and immunothrombosis, thereby obscuring mechanistic effects and weakening the linkage between experimental findings and clinical outcomes. In parallel, basic research has frequently examined injury mechanisms in isolated organs or signaling pathways, lacking a systemic and cross‐organ perspective. Existing reviews likewise tend to focus on individual organs or discrete mechanistic modules, without constructing a coherent logical framework linking core mechanisms, organ susceptibility, and clinical evidence. Collectively, these limitations underscore the urgent need for a comprehensive review that integrates cross‐organ common mechanisms, deeply interrogates the endothelial–immune–coagulation interaction network, and rigorously evaluates the evidence chain from mechanistic insight to clinical application.

Accordingly, this review delineates the pathobiological landscape of multiorgan IRI. We first summarize the major clinical scenarios, affected organ systems, and heterogeneous etiologies of IRI across different organs, thereby establishing the clinical and biological context. We then examine the core cellular and molecular mechanisms underlying IRI, including ischemia‐induced metabolic reprogramming and ionic homeostasis disruption, mitochondrial dysfunction‐driven oxidative stress, activation of regulated cell death (RCD) pathways (apoptosis, autophagy, and necroptosis), and the ensuing sterile inflammatory cascade. Particular emphasis is placed on the endothelial–immune–coagulation triadic axis, with detailed discussion of the important role of NETs in immune–thrombotic amplification and the positive feedback loops involving complement, platelets, and monocytes/macrophages. We further compare vulnerable structural units, organ‐specific NET phenotypes, and interorgan crosstalk pathways in major organs—including the liver, heart, brain, and kidney—to elucidate the determinants of organ‐specific IRI manifestations. In addition, we assess the translational fidelity of commonly used preclinical models in recapitulating these mechanisms and systematically review representative clinical evidence from myocardial reperfusion therapy, liver and kidney transplantation, and stroke recanalization, with a particular focus on recent advances in NETs‐ and immunothrombosis‐targeted interventions. Finally, based on an integrated synthesis of mechanistic insights and clinical evidence, we propose mechanism‐driven therapeutic principles and potential precision treatment strategies. By vertically integrating macroscopic clinical contexts with microscopic molecular mechanisms and horizontally synthesizing shared pathways with organ‐specific features, this review constructs a coherent framework for IRI pathophysiology. This framework provides a theoretical foundation for overcoming current translational barriers and for designing future clinical trials tailored to specific organs and pathological phenotypes. A sustained focus on the NET‐driven immunothrombotic network runs throughout this review, with the aim of advancing IRI research from fragmented, single‐organ paradigms toward an integrated, cross‐organ systems perspective.

## 2 Clinical and Pathological Framework of Multiorgan IRI

This section establishes the clinical and pathological framework of multiorgan IRI. It first outlines the clinical settings in which reperfusion aggravates injury, then explains determinants of heterogeneity and the modular sequence of pathogenic events. It also summarizes outcome metrics that link molecular mechanisms with organ‐specific endpoints.

### 2.1 Clinical Context and Organ Spectrum of IRI

IRI derives its clinical relevance from a fundamental paradox embedded in one of modern medicine's core therapeutic principles—restoration of tissue perfusion. With the rapid advancement of recanalization and reperfusion strategies, including thrombolysis, percutaneous coronary intervention, organ transplantation, and endovascular thrombectomy for acute ischemic stroke, IRI has transitioned from a theoretical construct to a major determinant of clinical prognosis across a wide range of diseases [11, 12]. It is now recognized as a pervasive pathological process spanning multiple disciplines, including cardiovascular medicine, neurology, transplantation, trauma, and critical care, and is characterized by a broad and systemic clinical spectrum [11].

Ischemic insults can arise in virtually any organ, whereas the pathological consequences of reperfusion frequently extend beyond the primary site of injury, resulting in both local tissue damage and dysfunction of distant organs. In cardiovascular medicine, myocardial IRI following successful coronary recanalization in patients with acute myocardial infarction can compromise myocardial salvage and precipitate malignant arrhythmias and contractile dysfunction, representing a major source of residual risk in the reperfusion era [13, 14]. In cerebrovascular disease, cerebral IRI after intravenous thrombolysis or mechanical thrombectomy is a key contributor to hemorrhagic transformation (HAT), cerebral edema, and neurological deterioration [15, 16]. Transplantation medicine provides a classical clinical context for IRI, as functional recovery of liver, kidney, lung, and intestinal grafts is inevitably constrained by cold ischemia during preservation and subsequent reperfusion, forming the pathological basis of primary graft dysfunction and delayed graft function (DGF) [17, 18].

In addition, IRI manifests as a systemic pathological event in conditions associated with global circulatory failure, such as shock resuscitation, cardiopulmonary bypass during cardiac surgery, post‐cardiac arrest syndrome, and mesenteric ischemia–reperfusion [19, 20]. In these settings, disruption of the intestinal barrier facilitates translocation of endotoxins and inflammatory mediators, driving systemic inflammation and the development of MODSs, including acute respiratory distress syndrome (ARDS) and acute kidney injury (AKI) [21, 22]. Collectively, these observations underscore that IRI is not a discrete disease entity but rather a common terminal pathway shared by diverse critical illnesses and therapeutic interventions. Its impact ranges from local tissue viability to global homeostatic failure, and appreciation of its pan‐organ spectrum constitutes the clinical foundation for a systematic understanding of IRI pathophysiology.

### 2.2 Determinants of Heterogeneity in IRI

Although IRI is governed by conserved biological mechanisms, its clinical severity, pathological manifestations, and outcomes exhibit marked heterogeneity across individuals and organs. This variability is not stochastic but instead reflects the combined influence of multiple determinants, including the characteristics of the ischemic insult, intrinsic properties of the affected organ, and the host's systemic physiological background.

First, features intrinsic to the ischemic event—particularly its duration and severity—are fundamental drivers of heterogeneity. Brief or partial ischemia may predominantly induce reversible metabolic adaptations and ischemic preconditioning, whereas prolonged or complete ischemia results in irreversible energy depletion and extensive cell death [23]. Consequently, the reperfusion‐associated injury pathways engaged, as well as their intensity, differ substantially between these scenarios. In the context of organ transplantation, cold ischemia associated with hypothermic preservation and warm ischemia resulting from normothermic interruption of blood flow elicit distinct molecular responses: cold ischemia preferentially promotes endothelial activation and microcirculatory dysfunction, whereas warm ischemia induces marked metabolic collapse [17].

Second, organ‐specific anatomical and metabolic characteristics constitute the structural basis of differential vulnerability [14]. Tissues with high oxygen demand, such as the myocardium and neurons, exhibit extreme sensitivity to hypoxia, and their limited regenerative capacity renders cell loss particularly detrimental. The liver's dual blood supply and abundance of resident macrophages (Kupffer cells) confer a prominent immunological and inflammatory signature to hepatic IRI. In the kidney, the corticomedullary oxygen gradient renders the thick ascending limb of the loop of Henle especially susceptible to ischemic stress. The lung, characterized by an extensive endothelial surface area, is highly prone to inflammatory cell sequestration and activation during systemic IRI.

Third, the host's systemic background critically modulates IRI susceptibility and progression [24]. Age‐related declines in cellular repair mechanisms, microvascular pathology, and chronic inflammation associated with comorbidities such as diabetes mellitus and atherosclerosis, and genetic polymorphisms affecting key injury and repair pathways collectively shape the baseline tolerance of tissues to ischemic stress and their reparative capacity [24]. In addition, the qualitative features of reperfusion—including perfusion pressure, oxygen tension, and the presence of persistent hypoperfusion or microcirculatory dysfunction—directly influence the ultimate extent of tissue injury.

Taken together, the heterogeneity of IRI arises from the dynamic interplay among injury signals within defined spatiotemporal contexts, organ‐specific microenvironments, and the host's systemic physiological state. Recognition of these multidimensional determinants highlights that effective therapeutic strategies must be tailored to this complexity, thereby providing the conceptual foundation for precision organ‐protective interventions.

### 2.3 Modular Hierarchy of Pathogenic Mechanisms

The pathogenic mechanisms of IRI do not unfold as linear events but instead operate as a hierarchical cascade composed of multiple dynamically interacting biological modules. The spatiotemporal coordination of these modules determines both the severity and reversibility of tissue injury. This hierarchy begins with the metabolic collapse module during ischemia. Interruption of oxygen supply rapidly depletes adenosine triphosphate (ATP), leading to sodium–potassium pump failure and subsequent cellular edema. Concurrently, anaerobic glycolysis results in lactate and proton accumulation, causing intracellular acidosis that primes tissues for reperfusion‐associated injury. The defining features of this phase are energy depletion and ionic homeostasis disruption, whose magnitude is governed by organ‐specific metabolic demand and ischemia duration.

Upon restoration of blood flow, the process transitions to the acute stress module, characterized by a burst of mitochondrial dysfunction. Calcium overload induces irreversible opening of the mitochondrial permeability transition pore (mPTP), resulting in collapse of the electron transport chain (ETC) and exponential generation of ROS and reactive nitrogen species (RNS). Simultaneously, uncoupling of nitric oxide synthases (NOSs) exacerbates nitrosative stress, directly damaging lipids, proteins, and DNA. This module serves as an important hub, perpetuating metabolic imbalance while activating downstream inflammatory and cell death pathways. Its intensity is dynamically regulated by reperfusion velocity and oxygen partial pressure.

Within hours, the RCD module becomes broadly activated, forming a multipathway “death network.” Canonical mitochondrial‐dependent apoptosis is regulated by the Bcl‐2 family, whereas necroptosis is mediated through the receptor‐interacting protein kinase (RIPK)1–RIPK3–mixed lineage kinase domain‐like pseudokinase (MLKL) axis. Pyroptosis depends on gasdermin D (GSDMD) pore formation downstream of caspase‐1/4/5 activation, while ferroptosis arises from lipid peroxidation driven by glutathione peroxidase 4 (GPX4) inactivation [25, 26]. These pathways do not function independently but instead amplify one another through shared mediators, such as mitochondrial DNA release [27, 28]. In highly vulnerable cell populations, including renal tubular epithelial cells and neurons, the predominance of specific death modalities is finely tuned by microenvironmental factors such as iron availability.

Release of DAMPs—including high‐mobility group box 1 (HMGB1), mitochondrial DNA, and extracellular ATP—rapidly activates the sterile inflammation module [29, 30]. Pattern recognition receptors (PRRs), such as Toll‐like receptor 4 (TLR4) and NLRP3 inflammasomes, initiate innate immune responses, promoting neutrophil recruitment, NET formation, and dual activation of the complement system via classical and alternative pathways [31, 32]. Complement fragment C5a further recruits monocytes and drives macrophage differentiation, leading to the release of proinflammatory cytokines such as tumor necrosis factor‐α (TNF‐α) and interleukin‐1β (IL‐1β) [33, 34]. This phase is marked by synergistic failure of the endothelial–immune–coagulation axis, manifested by increased microvascular permeability, platelet activation, and formation of an immunothrombotic microenvironment [35].

Over subsequent days to weeks, the tissue remodeling module becomes dominant, reflecting the balance between inflammatory resolution and repair. Persistent fibrosis is driven by transforming growth factor‐β (TGF‐β)‐activated myofibroblasts, whereas regenerative capacity is constrained by depletion or dysfunction of resident progenitor cells. The ultimate outcome of this phase depends on the severity of early injury and the host's intrinsic reparative potential.

This modular hierarchy reveals the internal logic of IRI pathogenesis: upstream modules fuel downstream responses (e.g., mitochondrial ROS activating NLRP3 inflammasomes), while organ‐specific features modulate the relative contribution of each module (e.g., disruption of tight junctions in cerebral microvascular endothelium accelerating edema formation). Understanding this hierarchical organization explains why interventions targeting single nodes—such as apoptosis inhibition alone—often fail, and it highlights the importance of time‐window‐specific strategies. For example, mPTP inhibition at the onset of reperfusion may interrupt the entire cascade, whereas late‐stage targeting of NETs can mitigate secondary immune–thrombotic injury. This framework provides a mechanistic foundation for subsequent analyses of organ vulnerability and therapeutic intervention strategies.

### 2.4 Clinical Outcome Metrics System

Evaluation of clinical outcomes in IRI requires moving beyond single‐organ functional measures toward a multidimensional, dynamically evolving metrics system that captures systemic pathological burden and long‐term prognosis. Organ‐specific endpoints constitute the core of this framework. In myocardial IRI, left ventricular ejection fraction (LVEF) and the extent of microvascular obstruction assessed by cardiac magnetic resonance imaging reflect structural and functional impairment, while cardiac enzyme profiles—such as high‐sensitivity troponin T—quantify cellular injury [36, 37]. Cerebral IRI is evaluated using neurological scales, including the National Institutes of Health Stroke Scale (NIHSS) and the modified Rankin Scale (mRS), in combination with diffusion‐weighted imaging lesion volume to differentiate reversible ischemic penumbra from irreversible infarction [38, 39]. In liver IRI, outcomes are frequently defined by early allograft dysfunction (EAD) criteria, integrating international normalized ratio (INR), peak bilirubin levels, and alanine aminotransferase (ALT) kinetics, while biliary complications such as ischemic cholangiopathy require long‐term monitoring by magnetic resonance cholangiopancreatography [40, 41]. Renal IRI assessment focuses on AKI staging according to Kidney Disease: Improving Global Outcomes (KDIGO) criteria, based on creatinine elevation and urine output, whereas emerging biomarkers such as neutrophil gelatinase‐associated lipocalin (NGAL) and the TIMP‐2/IGFBP7 combination provide early prediction of progression to chronic kidney disease (CKD) [42, 43]. In systemic settings, including trauma and transplantation, MODS scores—incorporating respiratory (PaO_2_/FiO_2_), circulatory, hepatic, and renal parameters—serve as robust predictors of intensive care unit (ICU) mortality [44].

Despite their utility, traditional endpoints exhibit important limitations. Functional indices such as LVEF often lag behind molecular injury and are confounded by baseline organ dysfunction, such as pre‐existing heart failure [45]. Consequently, biomarker panels require substantial innovation. Beyond conventional inflammatory markers (e.g., C‐reactive protein and IL‐6), emerging candidates—including circulating cell‐free DNA (cfDNA), complement fragments (e.g., C5a), and ferroptosis‐associated markers (e.g., ACSL4)—are being validated as mechanism‐specific indicators. Their temporal dynamics may enable discrimination among inflammatory subtypes, such as NET‐dominant versus complement‐driven responses.

Patient‐centered outcomes are gaining increasing prominence, encompassing cardiopulmonary performance metrics (e.g., 6‐min walk distance), health‐related quality‐of‐life assessments (SF‐36, EQ‐5D), and long‐term clinical events, such as heart failure readmission within 2 years following myocardial IRI [46, 47]. These measures capture the cumulative and often insidious impact of IRI on functional capacity and quality of life [48].

A major challenge in outcome assessment lies in indicator heterogeneity and limited standardization. Nonlinear relationships between cold ischemia time and graft outcomes in transplantation, as well as population‐dependent variability in the predictive performance of scores such as the HAT score after stroke thrombectomy, underscore the need for individualized risk stratification [49, 50]. Future outcome systems should integrate a three‐dimensional mechanism–organ–time framework: acute‐phase assessment of DAMP release and microcirculatory perfusion; subacute monitoring of immune–thrombotic biomarkers; and long‐term surveillance of fibrotic remodeling using indices such as the liver FIB‐4 score and renal estimated glomerular filtration rate (eGFR) slope [51, 52]. Such a multitiered metrics system not only enables precise endpoint selection for clinical trials but also facilitates mechanistic mapping between molecular pathways and clinical phenotypes. Through this approach, IRI management can progress from empirically driven supportive care toward mechanism‐based precision interventions, establishing quantifiable and actionable benchmarks for therapeutic development (Table 1).

**TABLE 1:** Organ‐specific clinical contexts, vulnerable structures, and outcome measures in ischemia–reperfusion injury.

Organ	Clinical setting	Vulnerable structures	Commonly used endpoints	References
Heart	Acute myocardial infarction with reperfusion; cardiac surgery‐related IRI	Cardiomyocytes; coronary microvascular endothelium; microvascular obstruction (MVO)	Infarct size, MVO, and IMH by CMR; LVEF; heart‐failure hospitalization; MACE and mortality	[53, 54, 55]
Brain	Acute ischemic stroke after recanalization	Ischemic penumbra neurons; blood–brain barrier; microvascular endothelium	NIHSS (acute); 90‐day mRS; infarct volume; symptomatic intracranial hemorrhage; mortality	[56, 57, 58]
Liver	Transplantation or major hepatectomy	Sinusoidal endothelial cells; hepatocytes; cholangiocytes and peribiliary vascular plexus	Early allograft dysfunction (AST/ALT, INR, bilirubin); L‐GrAFT score; graft failure or retransplantation; ICU stay	[59, 60, 61]
Kidney	Transplantation‐associated IRI; perioperative or shock‐related AKI	Proximal tubules (outer medulla); peritubular capillary endothelium; medullary microcirculation	Delayed graft function; serum creatinine and urine output; eGFR; graft survival; acute rejection	[62, 63, 64]
Lung	Lung transplantation reperfusion; ECMO weaning; ARDS phenotype	Alveolar–capillary barrier; pulmonary microvascular endothelium; alveolar Type II cells	Primary graft dysfunction grade; PaO_2_/FiO_2_; ventilator‐free days; ICU/28‐day mortality	[65, 66, 67]
Gut	Acute mesenteric ischemia; shock‐related intestinal IRI	Villus‐tip epithelium; tight‐junction barrier; mesenteric microcirculation	Bowel necrosis/resection rate; lactate; sepsis or MODS; mortality; CT angiographic findings	[68, 69]
Skeletal muscle	Crush injury; limb ischemia with revascularization	Myocytes and interstitium; compressed microvasculature; systemic release of myoglobin and potassium	Compartment syndrome; rhabdomyolysis (CK); hyperkalemia; AKI (KDIGO); mortality	[70]
Systemic (MODS)	Global ischemia–reperfusion after shock or resuscitation	Systemic endothelial–immune–coagulation axis; circulating DAMPs/NET‐related injury	SOFA score; organ‐support requirements; coagulation markers; circulating NET biomarkers (cfDNA, H3Cit)	[71]

Abbreviations: AKI, acute kidney injury; ALT, alanine aminotransferase; ARDS, acute respiratory distress syndrome; AST, aspartate aminotransferase; BBB, blood–brain barrier; cfDNA, cell‐free DNA; CK, creatine kinase; CMR, cardiovascular magnetic resonance; DAMPs, damage‐associated molecular patterns; ECMO, extracorporeal membrane oxygenation; eGFR, estimated glomerular filtration rate; H3Cit, citrullinated histone H3; ICU, intensive care unit; IMH, intramyocardial hemorrhage; INR, international normalized ratio; IRI, ischemia–reperfusion injury; KDIGO, Kidney Disease: Improving Global Outcomes; L‐GrAFT, Liver Graft Assessment Following Transplantation; LVEF, left ventricular ejection fraction; MACE, major adverse cardiovascular events; MODS, multiple organ dysfunction syndrome; mRS, modified Rankin Scale; MVO, microvascular obstruction; NETs, neutrophil extracellular traps; NIHSS, National Institutes of Health Stroke Scale; PaO2/FiO2, ratio of arterial oxygen partial pressure to fractional inspired oxygen; SOFA, Sequential Organ Failure Assessment.

## 3 Molecular and Cellular Mechanisms of IRI

**FIGURE 1:**
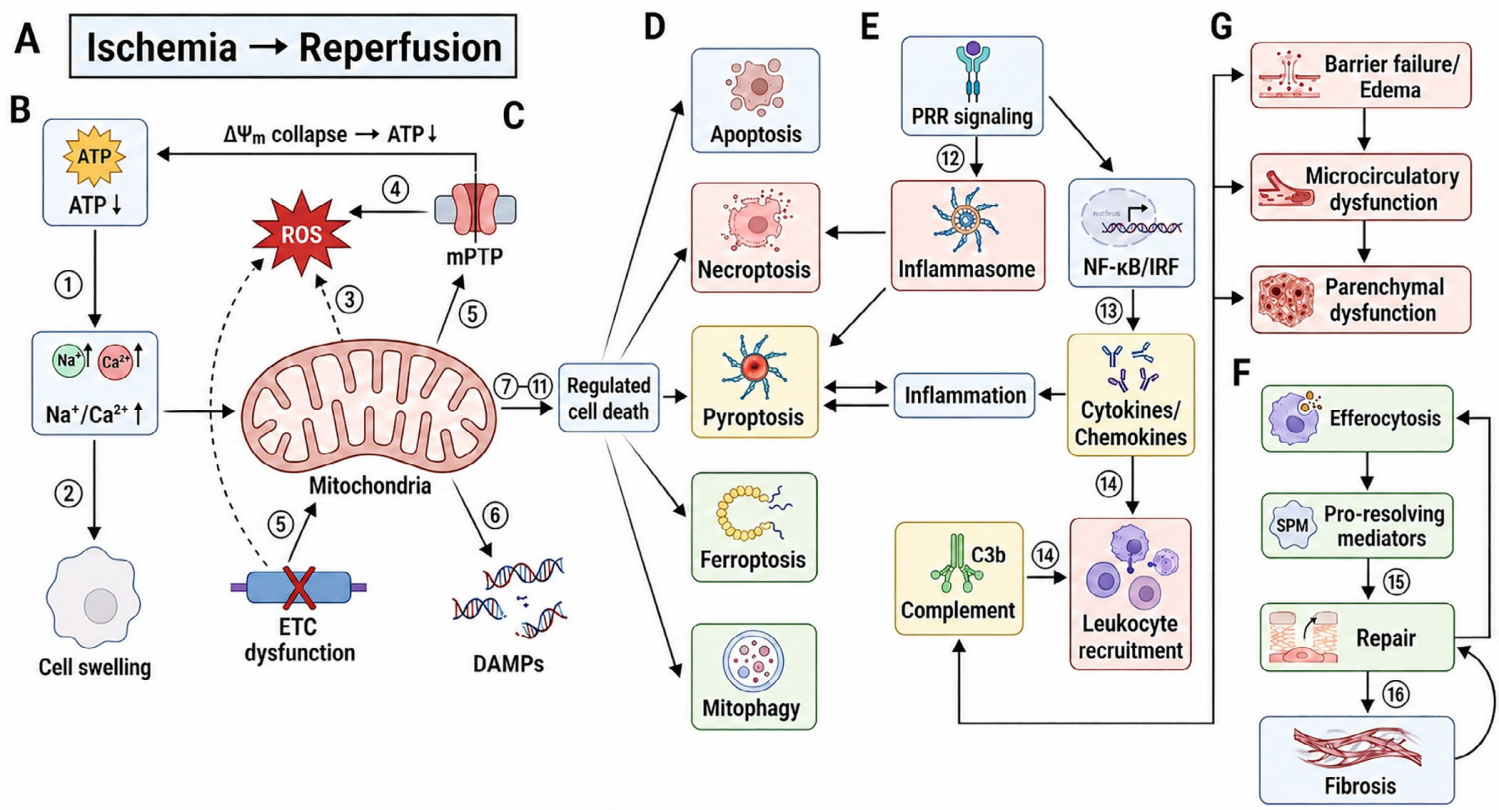
Mechanistic cascade of IRI integrating mitochondrial dysfunction, regulated cell death, sterile inflammation, and divergent tissue outcomes. Ischemia initiates ATP depletion and ionic disequilibrium (Na^+^/Ca^2^^+^ overload), leading to cellular swelling and priming mitochondrial injury (B and C). Upon reperfusion, mitochondrial electron transport dysfunction drives an early ROS burst and favors mitochondrial permeability transition pore (mPTP) opening, accelerating loss of membrane potential (ΔΨm) and further ATP decline, while promoting release of damage‐associated molecular patterns (DAMPs) (C). These upstream stresses converge on regulated cell death programs—apoptosis, necroptosis, pyroptosis, ferroptosis, and altered mitophagy/mitochondrial quality control (D). DAMPs activate PRR signaling and inflammasome pathways, engaging NF‐κB/IRF transcriptional programs to induce cytokines/chemokines, complement activation, and leukocyte recruitment, collectively amplifying sterile inflammation (E). Downstream, these processes culminate in barrier failure/edema, microcirculatory dysfunction, and parenchymal dysfunction (G). In parallel, a counter‐regulatory trajectory supports resolution through efferocytosis and specialized proresolving mediators, enabling repair; failure of resolution favors maladaptive remodeling and fibrosis (F). ATP, adenosine triphosphate; C3b, complement component 3b; DAMPs, damage‐associated molecular patterns; ETC, electron transport chain; IRF, interferon regulatory factor; mPTP, mitochondrial permeability transition pore; NF‐κB, nuclear factor kappa B; PRR, pattern‐recognition receptor; ROS, reactive oxygen species; SPM, specialized proresolving mediator; ΔΨm, mitochondrial membrane potential.

This section follows the injury cascade from metabolic stress to cell fate decisions and tissue remodeling. It begins with ATP depletion and ion imbalance, then discusses mitochondrial oxidative/nitrosative injury, RCD, sterile inflammation, and the transition from inflammation resolution to fibrosis or repair. The integrated sequence is summarized in Figure 1.

### 3.1 Metabolic Reprogramming and Ion Homeostasis

In IRI, metabolic reprogramming and disruption of ion homeostasis constitute the earliest and most decisive molecular events, establishing the foundation for subsequent injury cascades. During ischemia, interruption of oxygen delivery forces cells to shift abruptly from oxidative phosphorylation to anaerobic glycolysis [72]. Inhibition of the pyruvate dehydrogenase complex leads to pyruvate accumulation and its conversion to lactate, resulting in intracellular acidosis [73]. Concurrently, a sharp decline in ATP production causes failure of ATP‐dependent ion pumps. Inactivation of the Na^+^/K^+^‐ATPase results in intracellular Na^+^ accumulation, promoting reversal of the Na^+^/Ca^2^^+^ exchanger and inducing Ca^2^^+^ overload. Proton accumulation further exacerbates cellular edema through activation of the Na^+^/H^+^ exchanger.

Upon reperfusion, oxygen delivery is restored, yet energy regeneration remains delayed because of persistent metabolic enzyme dysregulation, including hexokinase inhibition and abnormal glycolytic flux. Impaired fatty acid β‐oxidation further limits mitochondrial substrate availability. Paradoxically, rapid correction of acidosis activates the Na^+^/H^+^ exchanger isoform 1 (NHE1), amplifying Na^+^ influx and secondary Ca^2^^+^ overload, thereby creating a self‐reinforcing cycle of ionic imbalance. These processes are strongly influenced by organ‐specific metabolic features: tissues with high oxygen demand, such as the myocardium and brain, are particularly vulnerable to ATP depletion, whereas the liver can partially buffer ischemic stress through glycogen reserves.

Ca^2^^+^ release from endoplasmic reticulum stress stores synergizes with mitochondrial Ca^2^^+^ uptake via the mitochondrial calcium uniporter, amplifying calcium signaling and activating Ca^2^^+^‐dependent proteases such as calpain, which directly disrupt cytoskeletal architecture and membrane integrity. Importantly, metabolic reprogramming is not merely a passive consequence of energy deprivation but also functions as an active signaling hub. Lactate accumulation suppresses cyclic AMP signaling via GPR81 receptors, modulating inflammatory gene expression, whereas succinate accumulation during ischemia fuels RET in early reperfusion, serving as a critical driver of mitochondrial ROS generation [74, 75].

Collectively, these interactions underscore the centrality of the metabolic–ionic network: ionic disturbances impair cellular volume regulation and signal transduction, while fluctuations in metabolic intermediates such as acetyl‐CoA and α‐ketoglutarate reshape epigenetic landscapes and influence transcriptional programs involved in injury and repair [76]. A detailed understanding of this module not only clarifies the initiation of IRI but also identifies actionable therapeutic targets [77]. For example, NHE1 inhibitors attenuate Ca^2^^+^ overload in myocardial IRI, whereas modulation of lactate dehydrogenase activity stabilizes intracellular pH in hepatic IRI [78]. Nevertheless, pronounced organ‐specific differences necessitate precision‐based intervention strategies. In summary, metabolic reprogramming and ion homeostasis disruption function as the “ignition switch” of the IRI cascade, directly precipitating mitochondrial dysfunction and oxidative stress and providing a logical entry point for understanding irreversible tissue injury.

### 3.2 Mitochondrial Dysfunction and Oxidative/Nitrosative Stress

As the major hub for cellular energy production and signaling integration, mitochondrial dysfunction represents an important inflection point at which IRI transitions toward irreversible injury [79]. Mitochondrial impairment markedly amplifies oxidative and nitrosative stress, thereby shaping cellular fate. During ischemia, progressive loss of mitochondrial membrane potential (ΔΨm) and dysfunction of ETC Complexes I and III lead to accumulation of reduced electron carriers, including NADH and FADH_2_. Upon reperfusion, reintroduction of oxygen in the setting of ETC disruption results in substantial electron leakage and massive generation of superoxide anions (O_2_^−^) at Complexes I and III. Succinate‐driven RET further intensifies these ROS bursts [80, 81].

Calcium overload serves as a primary trigger for sustained opening of the mPTP. This process depends on cyclophilin D, a cyclosporine A‐sensitive regulator, and leads to mitochondrial matrix swelling, outer membrane rupture, and release of proapoptotic factors such as cytochrome *c* and apoptosis‐inducing factor. The propensity for mPTP opening is strongly influenced by reperfusion kinetics: abrupt reperfusion accelerates pore opening through mechanical and oxidative stress, whereas controlled reperfusion may partially preserve mitochondrial integrity.

Oxidative stress is not confined to mitochondria but is amplified by cytosolic enzyme systems [82]. Xanthine oxidase generates ROS in the context of ischemia‐induced purine accumulation, while NADPH oxidases (NOX2 and NOX4) are activated by DAMPs [83]. At the same time, antioxidant defenses—including superoxide dismutases (SOD1/2) and GPX—are compromised by glutathione depletion, leading to lipid peroxidation (e.g., 4‐hydroxynonenal adducts), protein carbonylation, and DNA damage [84].

Nitrosative stress arises primarily from overexpression of inducible NOS during reperfusion, resulting in excessive nitric oxide (NO) production. NO rapidly reacts with superoxide to form peroxynitrite (ONOO^−^), a potent oxidant that nitrates tyrosine residues to generate 3‐nitrotyrosine, thereby inhibiting key enzymes such as manganese SOD and creatine kinase and further impairing energy metabolism [85]. Organ specificity is particularly evident at this stage: the high mitochondrial density of cardiomyocytes predisposes the heart to mPTP‐dependent injury, whereas the brain exhibits heightened vulnerability to nitrosative stress owing to limited antioxidant reserves.

In addition, excessive mitochondrial fission mediated by dynamin‐related protein 1, together with impaired mitophagy, hinders the clearance of damaged mitochondria and prolongs ROS production [86, 87]. These mechanisms are tightly interconnected through a self‐amplifying “ROS–Ca^2^^+^” feedback loop, in which mitochondrial ROS enhances ryanodine receptor activity and promotes endoplasmic reticulum Ca^2^^+^ release, further exacerbating mPTP opening [79]. Although interventions such as mPTP inhibitors and mitochondria‐targeted antioxidants (e.g., MitoQ) confer protection in preclinical models, clinical translation remains limited by narrow therapeutic windows and organ‐specific responses [88]. In summary, mitochondrial dysfunction and oxidative/nitrosative stress act as powerful amplifiers that convert initial metabolic disturbances into widespread molecular damage and activate multiple RCD pathways, underscoring the central importance of preserving mitochondrial homeostasis in interrupting the IRI cascade.

### 3.3 Regulated Cell Death

Within the molecular landscape of IRI, RCD has evolved from the classical apoptosis–necrosis dichotomy into a complex network of interrelated death programs characterized by concurrency, cross‐amplification, and intimate coupling with inflammation and microcirculatory dysfunction [89, 90]. Experimental models of IRI in the heart, brain, kidney, and liver consistently demonstrate that early reperfusion‐associated events—such as mitochondrial ROS bursts, Ca^2^^+^ overload, and metabolic substrate remodeling—not only directly initiate cell death but also propagate injury through DAMP release and innate immune activation [91, 92].

Against this backdrop, apoptosis, necroptosis, pyroptosis, ferroptosis, and autophagy/mitophagy together constitute a dynamic “cell death spectrum” that contributes to acute tissue injury, immune amplification, and long‐term remodeling in a time‐ and cell‐type‐dependent manner [89]. Increasing evidence indicates that RCD represents a key determinant of IRI heterogeneity: identical reperfusion conditions do not yield uniform cellular fate decisions. Instead, differences in metabolic infrastructure, microvascular architecture, and immune milieu across organs dictate which death pathways predominate, thereby shaping organ‐specific pathological and clinical phenotypes.

RCD does not occur in isolation; its crosstalk with sterile inflammation and immunothrombosis has emerged as a central theme in IRI research [93, 94]. Necroptosis and pyroptosis involve membrane rupture and release of inflammatory mediators, readily forming positive feedback loops with complement activation, coagulation cascades, and NET‐driven immunothrombosis [83]. Ferroptosis exacerbates inflammation through lipid peroxidation and membrane destabilization and can synergize with mitochondrial injury [95]. Autophagy and mitophagy exhibit context‐dependent, biphasic effects: early activation may be cytoprotective by limiting ROS production and DAMP leakage through clearance of damaged mitochondria, whereas sustained activation under severe stress can accelerate cell loss and tissue injury [83].

Accordingly, this section delineates the principal mechanisms and representative evidence for distinct RCD modalities in IRI, organized along the conceptual axis of “death modality–molecular hub–inflammatory and immune interactions–organ adaptability.” Collectively, these findings support a unifying view of cell death in IRI as a coordinated “programmed ecosystem” amenable to mechanistic stratification and evidence‐based targeting. The central scientific focus has thus shifted from whether cell death occurs to which death pathways dominate in specific cell types at defined time points and how they interact with inflammation and microcirculatory failure to determine outcomes. This perspective provides a continuous mechanistic foundation for subsequent discussion of sterile inflammation and interaction networks.

#### 3.3.1 Apoptosis

Apoptosis, the canonical form of programmed cell death, contributes substantially in the early stages of IRI. It is characterized morphologically by cell shrinkage, chromatin condensation, DNA fragmentation, and the formation of membrane‐bound apoptotic bodies. This tightly regulated process is generally considered anti‐inflammatory, as it limits the release of intracellular contents into the extracellular milieu.

In the context of IRI, apoptosis is primarily initiated through two major pathways: the intrinsic (mitochondrial) pathway and the extrinsic (death receptor‐mediated) pathway. The intrinsic pathway is activated by mitochondrial injury arising from the combined effects of ischemia‐induced energy depletion and reperfusion‐associated oxidative stress. Increased mitochondrial outer membrane permeability results in the release of cytochrome *c* into the cytosol, where it associates with apoptotic protease‐activating factor 1 and deoxyadenosine triphosphate to form the apoptosome. This multiprotein complex activates procaspase‐9, which subsequently triggers a proteolytic cascade involving the executioner caspases‐3 and ‐7. Activation of these caspases leads to cleavage of key structural and regulatory proteins, culminating in controlled cellular dismantling [96].

This pathway is tightly regulated by members of the B‐cell lymphoma 2 (BCL‐2) protein family. Proapoptotic proteins such as Bax and Bak promote mitochondrial outer membrane permeabilization, whereas antiapoptotic members, including BCL‐2 and BCL‐xL, counteract this process and preserve mitochondrial integrity. The extrinsic apoptotic pathway is initiated by ligand binding to death receptors on the plasma membrane, such as Fas (CD95) and TNF receptor 1 (TNFR1). Receptor engagement recruits the adaptor protein Fas‐associated death domain, leading to activation of caspase‐8, which then directly activates downstream executioner caspases or indirectly engages the mitochondrial pathway through Bid cleavage.

Although apoptosis is traditionally regarded as immunologically silent, this property is context dependent. In the inflammatory and hypoxic microenvironment of IRI, phagocytic clearance of apoptotic cells is often impaired, allowing apoptotic cells to progress to secondary necrosis. This secondary necrosis results in the release of DAMPs, thereby amplifying local and systemic inflammatory responses. Moreover, apoptosis shares upstream triggers—such as mitochondrial dysfunction, calcium overload, and ROS bursts—with other RCD pathways. As such, apoptosis may function as an early decision node within the broader cell death network, facilitating the transition toward more inflammatory modes of cell death, including necroptosis and pyroptosis. This positions apoptosis as a foundational component of the RCD landscape in IRI rather than an isolated or terminal event.

#### 3.3.2 Necroptosis

Necrotic apoptosis, more accurately termed necroptosis, is a regulated form of cell death governed by well‐defined molecular signaling pathways. Although its morphology resembles accidental necrosis—characterized by cellular swelling, plasma membrane rupture, and release of intracellular contents—it is a genetically programmed process that elicits a strong inflammatory response. In the context of IRI, necroptosis is frequently engaged when caspase‐8 activity within the apoptotic pathway is inhibited or insufficient, thereby functioning as an alternative or “backup” death program.

The core molecular machinery of necroptosis consists of the RIPK signaling axis, comprising receptor‐interacting protein kinase 1 (RIPK1), receptor‐interacting protein kinase 3 (RIPK3), and MLKL [97, 98]. Following stimulation by death receptor ligands, such as TNF‐α, and under tight ubiquitin‐dependent regulation, RIPK1 and RIPK3 interact through their RIP homotypic interaction motif domains to assemble a supramolecular complex known as the necrosome. Within this complex, RIPK3 phosphorylates MLKL, triggering a conformational change that promotes MLKL oligomerization and translocation to the plasma membrane or intracellular organelle membranes [99, 100]. Oligomerized MLKL forms membrane‐disrupting pores, leading to loss of membrane integrity, osmotic swelling, and ultimately cell lysis.

Accumulating evidence indicates that necroptosis plays a significant role in IRI across multiple organs [101]. In renal IRI, pharmacological inhibition or genetic disruption of key necroptotic kinases markedly attenuates tubular epithelial injury and preserves renal function [102]. Similarly, in myocardial IRI, necroptosis contributes to cardiomyocyte death and infarct expansion, while in hepatic IRI models, activation of this pathway correlates closely with hepatocellular injury and inflammatory infiltration [103, 104]. Beyond direct parenchymal cell loss, necroptosis amplifies tissue damage through the release of DAMPs, thereby intensifying sterile inflammation.

Accordingly, therapeutic strategies targeting components of the necroptotic pathway—such as RIPK1 inhibitors or MLKL‐directed interventions—have emerged as promising approaches for mitigating IRI. Continued investigation of necroptosis not only refines our understanding of regulated necrotic cell death under pathological conditions but also provides a mechanistic basis for developing anti‐inflammatory, tissue‐protective therapies.

#### 3.3.3 Pyroptosis

Pyroptosis is a highly proinflammatory form of RCD mediated by inflammatory caspases, including caspase‐1 and caspase‐4/5 in humans and caspase‐11 in mice [105, 106]. Its defining molecular event is the cleavage of GSDMD, which liberates the N‐terminal pore‐forming domain that oligomerizes within the plasma membrane to generate large, nonselective pores [107, 108]. This pore formation leads to rapid osmotic swelling and cell lysis, accompanied by the release of mature proinflammatory cytokines—most notably IL‐1β and IL‐18—as well as intracellular danger signals, thereby markedly amplifying local and systemic sterile inflammation.

Initiation of pyroptosis is typically dependent on inflammasome assembly [109, 110]. In IRI, DAMPs released from injured or necrotic cells—including extracellular ATP, mitochondrial DNA, and reactive oxygen species—are sensed by PRPs such as NLRP3 and AIM2 [111, 112]. These receptors recruit the adaptor protein apoptosis‐associated speck‐like protein containing a CARD and procaspase‐1, forming multiprotein inflammasome complexes. Activated caspase‐1 subsequently cleaves GSDMD to initiate membrane pore formation and processes pro‐IL‐1β and pro‐IL‐18 into their mature, secreted forms. In parallel, noncanonical inflammasome pathways are activated by cytosolic lipopolysaccharide, which directly engages caspase‐4/5 (human) or caspase‐11 (mouse), leading to GSDMD cleavage and pyroptotic cell death independently of caspase‐1 [113, 114].

Accumulating evidence demonstrates that pyroptosis contributes substantially to IRI across multiple organs, including the kidney, heart, brain, and liver [115, 116]. In renal IRI, activation of the NLRP3 inflammasome and caspase‐1‐dependent GSDMD cleavage are key drivers of tubular epithelial injury and acute kidney dysfunction [117, 118]. In myocardial IRI, pyroptosis in both cardiomyocytes and infiltrating immune cells exacerbates inflammatory amplification and expands infarct size [119, 120]. Similarly, in cerebral and hepatic IRI, pyroptotic signaling has been linked to blood–brain barrier disruption, hepatocellular injury, and worsening inflammatory cascades [121, 122]. Accordingly, therapeutic targeting of pyroptosis—using NLRP3 inhibitors, GSDMD antagonists, or IL‐1β–neutralizing strategies—has shown protective effects in multiple preclinical IRI models [123, 124].

Collectively, pyroptosis represents a mechanistic bridge between RCD and sterile inflammation. By directly coupling membrane rupture with cytokine release, it functions as a central amplifier of tissue injury in IRI, making its key molecular nodes attractive targets for therapeutic intervention [125].

#### 3.3.4 Ferroptosis

Ferroptosis is a distinct form of RCD characterized by iron dependence and the unchecked accumulation of lipid peroxides [126, 127]. Its biochemical and morphological features clearly differentiate it from apoptosis, necroptosis, and pyroptosis [128, 129]. The central mechanism of ferroptosis involves failure of intracellular antioxidant defense systems, resulting in peroxidation of polyunsaturated fatty acid‐containing phospholipids, particularly those enriched with arachidonic acid and adrenic acid [130, 131]. Accumulation of these toxic lipid hydroperoxides, when not efficiently reduced or eliminated, compromises membrane integrity and culminates in cell death.

A critical determinant of ferroptosis susceptibility is dysfunction of the cystine/glutamate antiporter system (system x_c^−^), which limits cellular cystine uptake and impairs glutathione synthesis. Loss or inactivation of GPX4, the key enzyme responsible for reducing lipid hydroperoxides, represents a central execution step of ferroptosis [132]. In the context of IRI, ischemia‐induced metabolic derangements and depletion of reduced coenzyme Q (CoQH_2_), together with reperfusion‐associated bursts of ROS, generate a pro‐oxidant environment that strongly favors ferroptotic signaling. Simultaneously, IRI is often accompanied by expansion of the labile intracellular iron pool, which catalyzes lipid peroxidation through Fenton chemistry and further accelerates membrane damage.

An expanding body of evidence implicates ferroptosis as a major contributor to IRI across multiple organs, including the kidney, heart, liver, and brain [133, 134]. In renal IRI, proximal tubular epithelial cells are particularly vulnerable due to their high metabolic demand and phospholipid membranes enriched in polyunsaturated fatty acids [135, 136]. Pharmacological inhibition of ferroptosis—using agents such as ferrostatin‐1, liproxstatin‐1, or iron chelators—has been shown to preserve tissue architecture and improve organ function in multiple preclinical models [137].

The transcription factor nuclear factor erythroid 2‐related factor 2 (NRF2) serves as a central endogenous regulator of ferroptosis resistance [138, 139]. NRF2 activation enhances cellular antioxidant capacity by upregulating genes involved in glutathione synthesis, iron sequestration, and detoxification pathways, including GPX4 and glutathione biosynthetic enzymes [140]. Owing to its unique biochemical underpinnings, ferroptosis represents a compelling therapeutic target in IRI, with potential for synergistic benefit when combined with interventions targeting other RCD pathways [141].

#### 3.3.5 Autophagy and Mitophagy

Autophagy and mitophagy function primarily as modulators of the IRI cell‐death spectrum rather than as unidirectional lethal programs [142, 143]. During early reperfusion, moderate autophagy and selective removal of damaged mitochondria can reduce sustained ROS production, limit mitochondrial DNA (mtDNA) leakage, and raise the threshold for cell death [144]. However, when lysosomal dysfunction impairs autophagic flux or disrupts the coupling between mitochondrial fission and clearance, accumulation of damaged organelles and oxidized lipids can drive cells toward apoptosis, necroptosis, or inflammatory lytic death [145].

Receptor‐mediated mitophagy pathways, including PINK1/Parkin‐ and FUNDC1‐dependent programs, have been repeatedly associated with susceptibility to ischemic injury in organs such as the kidney [146, 147]. Their regulation intersects with ATP/purine signaling, the mTOR axis, and inflammatory cues, indicating that indiscriminate “activation” or “inhibition” of autophagy may yield opposite outcomes depending on timing and cell type [148]. Thus, calibrating autophagic activity—rather than binary modulation—may represent a more effective therapeutic strategy.

In summary, RCD modalities—apoptosis, necroptosis, pyroptosis, ferroptosis, and autophagy/mitophagy—form a tightly interconnected network in IRI. These programs cross‐regulate one another; for example, caspase‐8 can initiate apoptosis while suppressing necroptosis, and ferroptosis is closely intertwined with mitochondrial dysfunction and oxidative stress. The emerging concept of “PANoptosis” further highlights molecular‐level integration among death pathways. Elucidating both the independent contributions and the interaction architecture of these programs is essential for designing interventions that modulate cell fate decisions in IRI. Future studies should prioritize mapping dynamic death‐network interactions across organs, cell types, and disease phases.

### 3.4 Initiation and Amplification of Sterile Inflammation

Sterile inflammation in IRI is not driven by infection but by an autonomously initiated cascade triggered by DAMPs [149]. Endogenous danger signals exceed tissue homeostatic thresholds, activate innate immune pathways, and can escalate into a systemic inflammatory response [150]. Cell death during ischemia and reperfusion releases DAMPs such as HMGB1, mtDNA, extracellular ATP, and heat shock proteins. With reperfusion, these mediators disseminate through the circulation and are sensed by PRRs—including TLR4 and the receptor for advanced glycation end products (RAGE)—expressed on macrophages, dendritic cells, and endothelial cells [151]. Downstream, TLR4/MyD88 signaling activates NF‐κB, promoting transcription of TNF‐α, IL‐6, and pro‐IL‐1β [152, 153]. In parallel, mitochondrial ROS, K^+^ efflux, or lysosomal disruption activates the NLRP3 inflammasome, which processes IL‐1β/IL‐18 via caspase‐1 and can induce pyroptosis, thereby reinforcing inflammatory amplification [154, 155].

Inflammation is further shaped by spatiotemporally coordinated immune‐cell recruitment. Neutrophils are among the earliest infiltrating cells; they adhere via integrin‐dependent interactions, release myeloperoxidase (MPO) and elastase, and exacerbate parenchymal injury. Monocytes differentiate into inflammatory macrophages and secrete chemokines such as CXCL1 and CCL2, recruiting additional effector cells. Complement activation amplifies neutrophil responses and NET formation through C5a–C5aR1 signaling [156]. Organ‐specific determinants are prominent: in cerebral IRI, blood–brain barrier disruption permits peripheral immune infiltration and activated microglia amplify neuroinflammation; in hepatic IRI, Kupffer cells dominate DAMP sensing; and in renal IRI, tubular epithelial cells can activate NLRP3, intensifying local inflammation [157].

NETs serve as important hubs in this amplification network. Neutrophil‐derived citrullinated histone H3 (CitH3) and neutrophil elastase (NE) can obstruct microcirculation and potentiate inflammatory signaling, including TLR9‐dependent responses to DNA‐containing complexes, while concurrently activating coagulation to form a self‐reinforcing immune–thrombotic cycle [158]. Host factors further modulate this axis: diabetes can augment RAGE signaling, whereas aging reduces regulatory T‐cell‐mediated suppression. Although interventions targeting this cascade (e.g., the TLR4 antagonist TAK‐242 and the NLRP3 inhibitor MCC950) reduce injury in preclinical models, clinical translation remains challenging because of tight coupling between inflammatory and coagulation pathways [159, 160].

In summary, initiation and amplification of sterile inflammation constitute a principal engine that escalates IRI severity. Its intensity and spatial reach are shaped by multireceptor coordination and organ microenvironmental context. These features not only define targets for resolution‐phase interventions but also highlight the time‐sensitive value of early blockade of the DAMP–PRR axis. If inflammation is not resolved promptly, it can drive progressive tissue destruction and naturally transitions into maladaptive repair and fibrosis.

### 3.5 Inflammation Resolution and Tissue Remodeling

Inflammation resolution is an active, orchestrated transition from tissue destruction to repair in IRI, driven by specialized proresolving mediators (SPMs) and immune‐cell phenotypic reprogramming. Its efficiency critically determines tissue fate: effective resolution supports regeneration, whereas failure promotes fibrosis and chronic organ dysfunction [161]. During late reperfusion, proinflammatory signals progressively give way to SPMs such as resolvin D1, lipoxin A4, and maresin 1, which act through receptors including ALX/FPR2 and GPR32 to suppress NF‐κB signaling, limit further neutrophil recruitment, and promote clearance of apoptotic cells [162].

Macrophages undergo a phenotypic transition from proinflammatory to reparative states and secrete mediators such as IL‐10, TGF‐β1, and vascular endothelial growth factor (VEGF), thereby promoting efferocytosis, stromal remodeling, and parenchymal repair. Tissue remodeling reflects a balance between parenchymal regeneration and stromal repair [163]. In the liver, regeneration is supported by Wnt/β‐catenin signaling; in the kidney, tubular repair can involve Sox9^+^ progenitor‐like cells; in the heart, limited regenerative capacity results in predominant scar formation [164, 165]. Persistent inflammation drives fibroblast activation via TGF‐β1, inducing myofibroblast differentiation and collagen deposition (e.g., Type I/III), culminating in fibrosis. These processes are microenvironment dependent—for example, hepatic stellate cell activation promotes perisinusoidal fibrosis, whereas renal tubulointerstitial fibrosis may involve epithelial–mesenchymal transition (Figure 1) [166].

Organ‐specific regenerative capacity strongly influences outcomes: limited neuronal regeneration and glial scar formation constrain recovery after cerebral IRI; alveolar Type II cells support substantial repair after pulmonary IRI, yet persistent inflammation can promote lung fibrosis; and skeletal muscle displays high reparative potential owing to abundant satellite cells. Phase‐specific interventions are therefore attractive but require spatiotemporal precision. Premature suppression of inflammation may impair debris clearance, whereas delayed resolution‐promoting therapy may miss the regenerative window [167, 168]. This module highlights that IRI prognosis depends not only on the magnitude of the initial injury but also on host resolution capacity. Biomarkers such as plasma resolvin D1 and urinary TGF‐β1 may help forecast repair trajectories, while personalized strategies will likely require integration of immune and metabolic reprogramming markers [169]. Ultimately, inflammation resolution and tissue remodeling represent the final link in the IRI feedback loop, determining long‐term organ function and emphasizing the dual therapeutic goals of injury containment and repair promotion (Table 2).

**TABLE 2:** Major regulated cell death pathways and representative evidence in ischemia–reperfusion injury.

Cell death pathway	Core mechanism	Key molecules	Representative evidence in IRI	Common experimental indicators	References
Mitochondrial apoptosis	Mitochondrial outer membrane permeabilization leads to cytochrome *c* release and caspase activation	BAX, BAK, cytochrome *c*, caspase‐3/9, BCL‐2 family	Proximal tubule‐specific Bax or Bak deletion reduces tubular apoptosis and improves renal function after renal IRI	DNA fragmentation (TUNEL); activated caspase‐3; cytosolic cytochrome *c*; apoptotic cell morphology	[170, 171]
Necroptosis	RIPK1–RIPK3 complex activates MLKL, causing membrane rupture when caspase‐8 is inhibited	RIPK1, RIPK3, MLKL	RIPK3–MLKL‐dependent necroinflammation promotes AKI‐to‐CKD transition following renal IRI	Phosphorylated MLKL; membrane rupture; LDH release; necrotic cell morphology	[172, 173, 174]
Pharmacologic inhibition of necroptosis	RIPK1 inhibition blocks downstream necroptotic signaling	RIPK1	Necrostatin‐1 treatment attenuates renal ischemia–reperfusion injury in experimental models	Reduced MLKL activation; smaller necrotic areas; improved organ function	[175, 176, 177]
Pyroptosis	Inflammasome activation cleaves gasdermin D, forming membrane pores and inducing inflammatory cell lysis	NLRP3, caspase‐1/4/5/11, gasdermin D	Genetic and mechanistic studies define gasdermin D pore formation as the executor of pyroptotic cell death	Gasdermin D cleavage; membrane pore formation; IL‐1β/IL‐18 release; cell swelling and lysis	[178, 179, 180]
Pyroptosis in renal injury	Gasdermin D‐mediated cell death amplifies inflammation and tissue injury	Gasdermin D	Gasdermin D‐deficient mice display altered susceptibility to acute kidney injury, highlighting its functional role	Changes in inflammatory cytokines; tissue injury scores; renal function indices	[181, 182, 183]
Ferroptosis	Iron‐dependent lipid peroxidation driven by GPX4 failure and oxidative stress	GPX4, SLC7A11, ACSL4, labile iron	Genetic inactivation of GPX4 triggers acute renal failure with characteristic ferroptotic features	Lipid peroxidation signals; mitochondrial shrinkage; loss of GPX4 activity	[184, 185, 186]
Ferroptosis in renal IRI	Ferroptosis is a major contributor to tubular injury and is therapeutically targetable	GPX4 axis, lipid peroxides	Pharmacologic inhibition of ferroptosis reduces tissue damage in renal ischemia–reperfusion models	Reduced lipid oxidative damage; improved renal histology and function	[187, 188, 189]
Autophagy/autosis	Dysregulated autophagy can shift from cytoprotection to autophagic cell death (autosis)	LC3, p62, Beclin‐1, Rubicon	Rubicon upregulation promotes autosis and worsens myocardial ischemia–reperfusion injury	Altered autophagic flux; accumulation of autophagosomes; tissue injury size	[190, 191, 192]
Autophagy in renal IRI	Basal and early autophagy supports cellular homeostasis during ischemic stress	ATG proteins, Beclin‐1 complex	Inhibition of autophagy exacerbates renal ischemia–reperfusion injury	Impaired clearance of damaged organelles; worsened renal function	[193, 194, 195]
Mitophagy	Selective removal of damaged mitochondria limits ROS production and DAMP release	PINK1, Parkin (PRKN), OPTN	PINK1–Parkin‐dependent mitophagy protects against renal ischemia–reperfusion injury	Mitochondrial clearance; reduced oxidative stress; preserved organ function	[196, 197, 198]
Integrated mitochondrial quality control	Coordination of apoptosis, ferroptosis, and autophagy through mitochondrial quality control	Mitochondrial QC regulators	Reviews synthesize mitochondrial quality control as a unifying therapeutic axis in IRI	Integrated assessment of mitochondrial damage, oxidative stress, and cell survival	[199, 200, 201]

Abbreviations: ACSL4, acyl‐CoA synthetase long‐chain family member 4; AKI, acute kidney injury; ATG, autophagy‐related gene; BAK, BCL‐2 antagonist/killer; BAX, BCL‐2‐associated X protein; BCL‐2, B‐cell lymphoma 2; CKD, chronic kidney disease; DAMPs, damage‐associated molecular patterns; GPX4, glutathione peroxidase 4; IL, interleukin; IRI, ischemia–reperfusion injury; LC3, microtubule‐associated protein 1 light chain 3; LDH, lactate dehydrogenase; MLKL, mixed lineage kinase domain‐like protein; NLRP3, NOD‐like receptor family pyrin domain containing 3; OPTN, optineurin; PINK1, PTEN‐induced kinase 1; PRKN, parkin RBR E3 ubiquitin protein ligase; RIPK1, receptor‐interacting protein kinase 1; RIPK3, receptor‐interacting protein kinase 3; ROS, reactive oxygen species; SLC7A11, solute carrier family 7 member 11; TUNEL, terminal deoxynucleotidyl transferase dUTP nick end labeling.

## 4 Endothelial–Immune–Coagulation Crosstalk and Immunothrombosis

**FIGURE 2:**
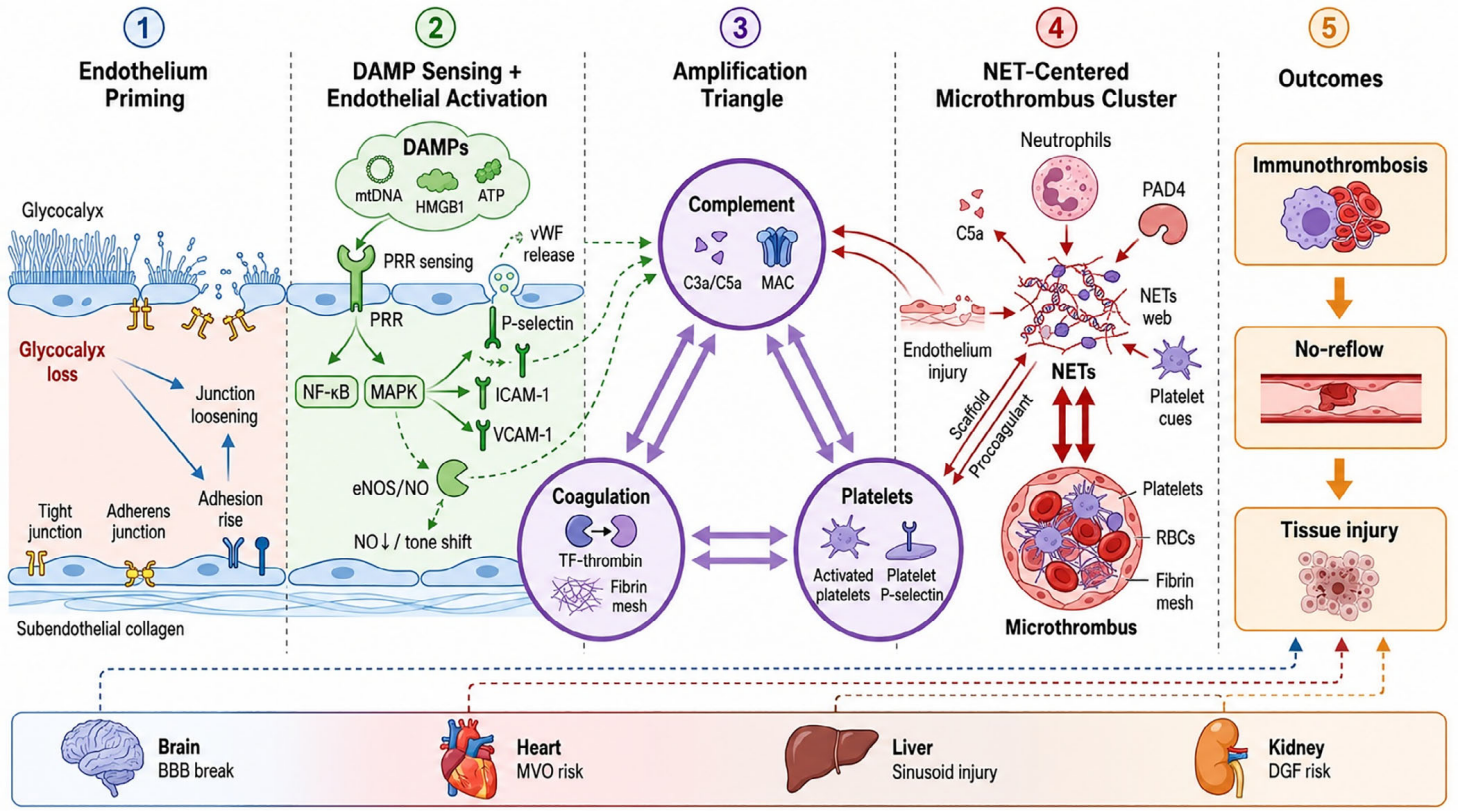
Immunothrombosis interaction network in ischemia–reperfusion injury: NET‐centered amplification across endothelium, complement, and coagulation. IRI provokes endothelial activation and barrier disruption, promoting leukocyte adhesion and a procoagulant shift that initiates platelet activation and fibrin‐rich microthrombi. Complement activation amplifies this response by enhancing endothelial injury, platelet/coagulation activity, and myeloid recruitment. NETs act as a central hub, providing a DNA–histone scaffold that traps platelets and coagulation factors while directly injuring endothelium; in turn, platelet signaling, complement (e.g., C5a), inflammatory mediators, and hypoxia promote NET formation, creating self‐reinforcing feedback loops. Monocytes/macrophages and adaptive immune remodeling further modulate amplification versus resolution. These convergent pathways culminate in microvascular obstruction (“no‐reflow”), tissue hypoxia, and organ dysfunction. The network also highlights clinically tractable readouts (e.g., NET, endothelial, coagulation, and complement biomarkers) and therapeutic nodes (NET targeting, complement blockade, endothelial protection, and time/phenotype‐guided antithrombotic strategies) while emphasizing bleeding–infection trade‐offs. ATP, adenosine triphosphate; BBB, blood–brain barrier; C3a/C5a, complement components 3a/5a; DAMPs, damage‐associated molecular patterns; DGF, delayed graft function; eNOS, endothelial nitric oxide synthase; HMGB1, high‐mobility group box 1; ICAM‐1, intercellular adhesion molecule 1; MAC, membrane attack complex; MAPK, mitogen‐activated protein kinase; mtDNA, mitochondrial DNA; MVO, microvascular obstruction; NETs, neutrophil extracellular traps; NF‐κB, nuclear factor kappa B; NO, nitric oxide; PAD4, peptidyl arginine deiminase 4; PRR, pattern‐recognition receptor; RBCs, red blood cells; TF, tissue factor; VCAM‐1, vascular cell adhesion molecule 1; vWF, von Willebrand factor.

This section focuses on vascular mechanisms that determine whether restored macroscopic flow results in effective tissue perfusion. It first describes endothelial activation and glycocalyx loss, then explains how complement, coagulation, platelets, and neutrophils converge on NET‐centered immunothrombosis [202, 203]. These interactions are summarized in Figure 2.

### 4.1 Endothelial Activation and Microcirculatory Dysfunction

As the principal interface between the vascular barrier and immune regulation, endothelial activation represents an early initiating event in microcirculatory failure during IRI. By dynamically reshaping vascular permeability, adhesion molecule expression, and vasomotor tone, activated endothelium directly determines the adequacy of tissue reperfusion. During ischemia, ATP depletion promotes cytoskeletal disorganization and internalization or destabilization of junctional proteins (e.g., VE‐cadherin and occludin), thereby weakening barrier integrity. Upon reperfusion, abrupt ROS generation accelerates glycocalyx degradation, exposing subendothelial matrix components and procoagulant mediators (including collagen and tissue factor [TF]) and facilitating activation of the coagulation cascade.

Concurrently, DAMPs engage TLRs (TLR2/4) and activate NF‐κB and MAPK signaling, inducing endothelial expression of E‐selectin, ICAM‐1, and VCAM‐1 and promoting leukocyte rolling, firm adhesion, and transendothelial migration. These responses are shaped by organ‐specific microvascular architecture [204, 205]. Brain microvascular endothelium exhibits highly restrictive barrier properties due to dense tight junctions; however, reperfusion‐associated permeability surges—often mediated by VEGF—can rapidly precipitate vasogenic edema. In the liver, sinusoidal endothelial cells lack a classical basement membrane and display fenestrations, rendering them particularly susceptible to mechanical and oxidative stress; TNF‐α released by Kupffer cells can further promote endothelial detachment and worsen intrahepatic microcirculatory resistance [206]. In the kidney, glomerular endothelium, adapted for high filtration, is especially vulnerable to complement‐mediated injury.

A hallmark of microcirculatory failure is the “no‐reflow” phenomenon, in which capillary networks remain inadequately perfused despite successful recanalization of upstream vessels, driven by endothelial swelling, leukocyte plugging, and microthrombus formation. Endothelial dysfunction extends beyond physical barrier disruption to encompass dysregulation of vasoactive and hemostatic signaling. Reduced NO bioavailability—often linked to endothelial NOS uncoupling—together with increased endothelin‐1 (ET‐1) release promotes vasoconstriction, while imbalance of prostacyclin versus thromboxane A_2_ favors platelet activation and aggregation.

Preclinical interventions aimed at restoring endothelial integrity—such as angiopoietin‐1–based approaches for glycocalyx repair or the Rho kinase inhibitor fasudil—have improved microvascular perfusion in animal models. However, translation to humans is limited by endothelial heterogeneity and variable reparative capacity; for example, diabetes‐associated endothelial progenitor dysfunction may impair vascular repair. Conceptually, endothelial activation is best viewed as an integrative node within the vascular–immune–coagulation axis [207, 208]. The severity of endothelial injury not only predicts local tissue necrosis but also promotes systemic inflammation and coagulopathy through the release of mediators such as von Willebrand factor (vWF) and TF. This provides a mechanistic foundation for the downstream complement–coagulation–platelet amplification axis and underscores the strategic value of preserving endothelial homeostasis to disrupt the self‐perpetuating cycle of IRI.

### 4.2 Complement–Coagulation–Platelet Amplification Axis

Complement activation, coagulation, and platelet signaling form a tightly coupled amplification axis in IRI that converts localized tissue injury into systemic immunothrombotic pathology through reciprocal molecular reinforcement [209]. Complement activation frequently acts as an upstream driver [209]. During ischemia, externalization of phosphatidylserine on stressed cell membranes can facilitate C1q binding and initiate the classical pathway. During reperfusion, mannose‐binding lectin (MBL) recognition of altered glycosylation patterns on injured endothelium can trigger the lectin pathway, whereas the alternative pathway may be sustained by damaged surfaces and mitochondrial‐derived components that stabilize C3 convertase activity.

Generation of the anaphylatoxins C3a and C5a engages C3aR and C5aR1, promoting endothelial activation (including P‐selectin upregulation) and neutrophil degranulation with release of NE [210, 211]. NE can further potentiate complement activation by cleaving C5 and increasing C5a availability, thereby creating a feed‐forward loop. In parallel, insertion of the C5b‐9 membrane attack complex (MAC) into endothelial membranes promotes procoagulant remodeling, including TF exposure. These events converge on coagulation initiation: TF forms a complex with factor VIIa (FVIIa), activates factor X (FX), and promotes thrombin generation [212]. Thrombin not only catalyzes fibrin formation but also signals via protease‐activated receptors (PARs) on platelets, endothelial cells, and monocytes, thereby amplifying inflammatory programs and inducing mediators such as IL‐6, IL‐8, and CD40L.

Platelets function as a major hub within this axis [213]. Thrombin and ADP drive platelet activation and surface P‐selectin expression, enabling platelet–neutrophil aggregate formation [214, 215]. Activated platelets can also release HMGB1 and mitochondrial DNA, which further stimulate complement pathways and inflammasome signaling (including NLRP3) [216]. Organ‐specific patterns have been described, including GPVI‐dependent platelet–collagen interactions contributing to microthrombus formation in myocardial IRI and MBL‐driven complement activation during cold ischemia in liver transplantation, where circulating sC5b‐9 levels have been associated with early graft dysfunction [217, 218]. In sepsis‐associated IRI, alternative pathway dominance has been reported in some models, and genetic or pharmacologic disruption of factor B has been linked to protection in renal injury settings [219].

The amplification potency of this axis reflects multiple interlocking positive feedback loops: thrombin can enhance complement activity, C5a can increase TF expression, and platelet TLR4‐mediated sensing of DAMPs can promote release of polyphosphates that accelerate contact pathway activation (e.g., factor XII). Clinical translation is constrained by a trade‐off between efficacy and safety [220, 221]. Broad anticoagulation (e.g., heparin) increases bleeding risk, whereas more selective strategies, such as C5 blockade with eculizumab, have shown signals of benefit in some renal IRI contexts but have not consistently improved outcomes in myocardial infarction trials (e.g., COMPASS), emphasizing organ‐ and phase‐specific therapeutic windows [222, 223].

Overall, complement–coagulation–platelet crosstalk does not represent parallel pathways but rather a self‐reinforcing network that can exponentially amplify initial DAMP signaling. This conceptual framework also supports positioning NETs as downstream effector hubs: NET formation is promoted by this axis, and NETs in turn reinforce complement activation, platelet recruitment, and coagulation, transforming localized microthrombosis into systemic immunothrombotic phenotypes [224].

### 4.3 Neutrophils and NETs: The Immunothrombotic Hub

Within the vascular microenvironment of IRI, NETs are major effectors that link inflammation to coagulation and can convert protective hemostasis into occlusive immunothrombosis [225, 226]. During early reperfusion, endothelial activation, disturbed shear stress, and DAMP release promote selectin‐mediated neutrophil rolling, integrin‐dependent adhesion, degranulation, and reactive oxygen species‐dependent chromatin decondensation [227]. Peptidylarginine deiminase (PAD)‐mediated histone citrullination relaxes chromatin and supports NET scaffold formation, while activated platelets amplify NET release through direct platelet–neutrophil contact and soluble mediators [228, 229].

Once deposited in the vascular lumen, NETs promote injury through structural obstruction and molecular cytotoxicity [230, 231]. The DNA–histone scaffold supports platelet adhesion, fibrin deposition, and contact pathway activation, whereas NET‐associated histones and proteases damage the endothelial glycocalyx and plasma membrane [232]. These processes reinforce a procoagulant and barrier‐disruptive phenotype, resulting in no‐reflow, microthrombus formation, and tissue edema.

NETs also create positive feedback with complement, platelets, and endothelial cells [233, 234]. Complement cleavage products promote mitochondrial ROS generation and NET release, while terminal complement complexes expand procoagulant endothelial surfaces and facilitate platelet adhesion. This feedback loop explains why NET‐associated immunothrombosis can persist after macroscopic vessel reopening and why it appears across organs despite differences in microvascular structure and resident immune composition.

From a translational perspective, NETs are attractive because they are measurable in tissue and blood. Tissue NET burden is commonly inferred from DNA colocalization with NE, MPO, or citrullinated histone H3, whereas circulating cfDNA, MPO–DNA complexes, and citrullinated histone H3 are used as surrogate markers [235]. These biomarkers are most useful for longitudinal tracking and within‐study stratification because extracellular DNA sources and assay methods vary across cohorts.

Clinically, NET‐associated signals have been linked to microvascular obstruction and early adverse outcomes after reperfusion in myocardial infarction and to thrombus organization and thrombolysis resistance in ischemic stroke [236, 237]. Placing NETs at the center of endothelial–immune–coagulation crosstalk therefore integrates inflammatory amplification, microcirculatory dysfunction, and coagulation imbalance into a testable framework (Figure 2). Therapeutic strategies should combine biomarker‐guided patient selection with careful timing, because suppressing pathological intravascular NET formation must be balanced against preservation of antimicrobial defense.

## 5 Organ Vulnerability and Systemic Crosstalk

This section compares how conserved IRI modules are shaped by organ‐specific microanatomy and resident immune environments. The liver, heart, brain, kidney, lung, gut, and skeletal muscle are discussed as distinct but interconnected targets [238, 239]. Particular attention is given to interorgan spillover that converts local reperfusion injury into systemic inflammation and multiple organ dysfunction.

### 5.1 Liver and Biliary Tract IRI

IRI of the liver and biliary tract is of major clinical relevance in liver transplantation, hepatectomy, and severe shock resuscitation [240, 241]. Rather than representing simple transient hepatocyte necrosis, hepatic IRI is a temporally and spatially heterogeneous process driven by coordinated dysfunction of hepatic sinusoidal endothelial cells (LSECs), hepatocytes, biliary epithelium, and the innate immune network [242, 243]. The liver's unique dual blood supply and specialized sinusoidal architecture confer exceptional sensitivity to alterations in perfusion patterns and oxygen availability. LSECs lack a continuous basement membrane, contain fenestrations, and are directly exposed to mixed portal venous and hepatic arterial blood flow.

During ischemia, cellular energy depletion induces cytoskeletal reorganization and internalization of junctional proteins in LSECs. Upon reperfusion, bursts of reactive oxygen and nitrogen species (ROS/RNS) rapidly damage the endothelial glycocalyx and plasma membranes, promoting endothelial detachment and exposure of the sinusoidal basement matrix. This enables direct contact of platelets, erythrocytes, and neutrophils with collagen and TF, initiating focal immunothrombosis [244, 245]. Early endothelial dysfunction thereby establishes the structural basis for the hepatic “no‐reflow” phenomenon, explaining the clinical paradox in which microcirculatory perfusion remains inadequate despite restoration of portal and arterial inflow.

Concurrently, hepatocytes undergo marked metabolic reprogramming during ischemia. Accumulation of tricarboxylic acid cycle intermediates—particularly succinate—primes mitochondria for exaggerated RET and oxidative stress upon reperfusion [246, 247]. Impaired fatty acid β‐oxidation and collapse of mitochondrial membrane potential synergistically enhance lipid peroxidation and iron‐dependent cell death pathways [248]. This metabolic–mitochondrial vulnerability is especially pronounced in steatotic donor livers, accounting for their heightened sensitivity to cold ischemia and reperfusion and the associated increased risk of early graft dysfunction and biliary complications [249].

At the immunological level, the hepatic macrophage compartment, dominated by Kupffer cells, functions both as an amplifier and a regulatory node during IRI. Kupffer cells sense danger‐associated molecular patterns released from injured hepatocytes and mitochondria via PRPs, rapidly producing TNF‐α, ILs, and chemokines that recruit neutrophils and monocytes [250]. They also activate inflammasomes, driving cytokine maturation and pyroptotic signaling [251]. Excessive Kupffer cell activation, however, markedly enhances the procoagulant milieu within sinusoids through TF expression and microparticle release, converting sterile inflammation into sustained immunothrombosis [252]. Persistent sinusoidal obstruction and hypoperfusion, particularly within centrilobular zones, promote the transition from potentially reversible cellular stress to irreversible necrosis and fibrogenesis.

NETs have emerged as key effectors in hepatic and biliary IRI [253, 254]. During early reperfusion, neutrophils are readily retained within the low‐shear sinusoidal environment and, under the influence of complement activation, platelet interactions, and endothelial adhesion signals, undergo NET formation [255]. These extracellular chromatin networks—enriched in histones, MPO, and elastase—mechanically obstruct microvessels and directly injure sinusoidal endothelium and pericholangiolar microvessels through histone‐mediated membrane damage [256, 257]. As a result, cholangiocytes are exposed to persistent hypoxia and inflammation [258].

The biliary epithelium is particularly vulnerable because it relies almost exclusively on small branches of the hepatic artery for perfusion and therefore exhibits a low ischemic tolerance threshold. During reperfusion, immune thrombi, endothelial edema, and microvascular compression within the pericholangiolar plexus precipitate progressive ischemic cholangitis [259]. This process is characterized by biliary epithelial apoptosis, luminal debris accumulation, and subsequent fibrotic stricture formation, clinically manifesting as persistent hyperbilirubinemia, biliary dilation, bile leakage, and recurrent cholangitis. These lesions constitute a major determinant of poor long‐term outcomes following liver transplantation, especially in grafts from donation after brain death or circulatory death donors [260, 261].

Preservation and reperfusion modalities further shape the spectrum of hepatic and biliary injury [262, 263]. Static cold storage suppresses metabolic activity but permits accumulation of metabolic intermediates and DAMPs during ischemia, resulting in a synchronized burst of mitochondrial dysfunction, endothelial injury, and NET formation upon rewarming and reperfusion [264]. In contrast, hypothermic or normothermic machine perfusion enables gradual restoration of oxygen delivery, clearance of metabolic waste and DAMPs, and preservation of endothelial glycocalyx integrity [265, 266]. Importantly, these platforms also provide an ex vivo therapeutic window for targeted delivery of complement inhibitors, NET‐degrading agents, or mitochondrial protective compounds [267].

Clinically, combined perioperative changes in circulating cfDNA, citrullinated histones, and biliary injury markers correlate closely with early graft dysfunction, ischemic cholangiopathy, and long‐term fibrotic remodeling [268, 269]. These observations support the development of stratified management strategies centered on sinusoidal endothelial injury, NET burden, and biliary microcirculatory integrity. Collectively, hepatic and biliary IRI represents a tightly coupled, organ‐specific network integrating metabolic dysfunction, mitochondrial injury, endothelial damage, immune activation, and coagulation [270, 271]. The dual vulnerability of sinusoidal endothelium and biliary epithelium defines both the frontline of early microcirculatory failure and the origin of chronic biliary remodeling and portal hypertension. Advancing mechanistic understanding of this network—together with machine perfusion platforms, organoid systems, and multimodal biomarkers—will facilitate a transition from empirical donor selection and perioperative management toward precision organ‐protective strategies guided by microcirculatory and immunothrombotic phenotypes [272].

### 5.2 Myocardial IRI

Myocardial IRI is the central pathological process underlying reperfusion therapy for acute myocardial infarction and exerts a decisive influence on heart failure progression and patient survival [273, 274]. Myocardial vulnerability reflects the exceptionally high metabolic demands of cardiomyocytes and their specialized microcirculatory architecture [275, 276]. Cardiomyocytes contain an extremely high density of mitochondria, yet possess limited glycolytic reserve; consequently, ischemia rapidly depletes ATP and induces failure of the Na^+^/K^+^‐ATPase. Upon reperfusion, Ca^2^^+^ overload synergizes with ROS bursts to trigger irreversible opening of the mPTP, leading to cardiomyocyte apoptosis and regulated necrotic cell death [277, 278].

At the microvascular level, myocardial IRI is characterized by the “no‐reflow phenomenon,” in which endothelial glycocalyx degradation exposes subendothelial collagen, promoting platelet activation and formation of platelet–neutrophil aggregates [279, 280]. NETs assemble into DNA–fibrin networks within the coronary microcirculation, mechanically obstructing perfusion while releasing histone H4, which directly disrupts cardiomyocyte membrane integrity [281, 282]. Organ‐specific mechanisms further reflect disruption of energy–electrical coupling: degradation of gap junction proteins impairs electrical conduction and predisposes to malignant arrhythmias, while macrophage‐derived advanced glycation end products amplify IL‐1β‐driven pyroptotic signaling [283]. NET‐associated oxidants additionally modify sarcoplasmic reticulum Ca^2^^+^‐handling proteins, exacerbating contractile dysfunction [284, 285].

Clinically, ischemic conditioning strategies—such as transient reocclusion during reperfusion—attenuate mPTP opening, although therapeutic efficacy is limited by a narrow temporal window [286, 287]. In preclinical studies, PAD4 inhibitors targeting NET formation improve myocardial microcirculation, and clinical observations demonstrate correlations between circulating CitH3 levels and adverse left ventricular remodeling [288, 289]. The distinctive feature of myocardial IRI lies in the fragility of electromechanical coupling and the heart's limited regenerative capacity [290, 291]. NET‐driven immunothrombosis in the acute phase not only determines infarct size but also initiates systemic inflammatory responses through release of myocardium‐specific DAMPs, thereby contributing to distant organ injury [292, 293]. These findings emphasize the need to integrate local microcirculatory repair with systemic immune modulation to achieve effective myocardial protection [294, 295].

### 5.3 Cerebral IRI

Cerebral IRI is a major determinant of disability and complications after reperfusion therapy for ischemic stroke [296, 297]. Its pathobiology reflects two intersecting vulnerabilities: the fragility of the neurovascular unit and the limited reserve of the blood–brain barrier (BBB) [298, 299]. During large‐vessel occlusion, microvascular endothelial cells undergo energy failure–driven cytoskeletal reorganization and internalization of tight‐junction proteins [300]. Upon reperfusion, abrupt changes in shear stress and bursts of oxidative stress activate matrix metalloproteinases—particularly MMP‐9—leading to degradation of basement membrane components and junctional complexes. The resulting barrier breakdown promotes vasogenic edema, characterized by interstitial fluid accumulation and rapid expansion of the edematous penumbra.

Astrocytic endfeet are highly sensitive to ischemia and inflammatory signaling. Remodeling of water and ion channels (e.g., aquaporin‐4 (AQP4) and inwardly rectifying potassium channel 4.1 (Kir4.1)) can exacerbate edema and disrupt potassium buffering and glutamate clearance, thereby amplifying excitotoxic injury. In parallel, microglia—the resident innate immune sentinels of the brain—rapidly transition from a ramified to an activated phenotype during early reperfusion. Through recognition of neuron‐derived DAMPs and blood‐borne cues via TLRs (TLR2/4), microglia activate NF‐κB and inflammasome pathways and release IL‐1β, TNF‐α, and chemokines that promote neutrophil recruitment and BBB transmigration.

Sustained NLRP3 inflammasome activation, driven by ionic disequilibrium and mitochondrial damage signals, further couples inflammation to barrier disruption [301, 302]. Caspase‐1‐mediated GSDMD cleavage induces membrane pore formation in endothelial cells and neurons, linking pyroptosis with BBB failure. Recruited neutrophils undergo NETosis in response to locally elevated DAMPs, complement fragments, and platelet‐derived signals, releasing DNA‐based NET scaffolds decorated with MPO and elastase [303, 304]. Citrullinated histones and proteases within NETs directly injure endothelial cells and astrocytic endfeet, widening BBB gaps and facilitating erythrocyte extravasation and HAT [305]. In addition, NETs provide a procoagulant surface that concentrates platelets and coagulation factors, promoting immunothrombi within microvascular lumens and producing a spatial dissociation between successful large‐vessel recanalization and effective tissue reperfusion.

Neurons are intrinsically vulnerable to reperfusion stress. High mitochondrial density and limited antioxidant capacity predispose neurons to mitochondrial membrane potential collapse and mPTP opening during peak ROS exposure. Release of mitochondrial DAMPs and cytochrome *c* activates apoptotic cascades, whereas impaired glutamate reuptake sustains calcium overload and dysregulated calmodulin‐dependent signaling. In the setting of perturbed iron handling, lipid peroxidation is enhanced and ferroptotic death programs may be engaged, contributing to a multipathway cascade of neuronal loss. Oligodendrocytes exhibit an even lower tolerance to oxidative and energetic stress; their injury compromises myelin integrity, limits remyelination, and thereby influences long‐term white matter remodeling and functional recovery.

Clinically, these cellular and molecular events correspond to imaging and outcome phenotypes such as contrast extravasation, edema expansion, delayed HAT, and early neurological deterioration. Worsening NIHSS scores soon after reperfusion often coincide with early BBB disruption and elevated immunothrombotic burden [306]. Consistently, NET‐related biomarkers and inflammatory mediators in cerebrospinal fluid or plasma associate with HAT, malignant cerebral edema, and poor prognosis, underscoring dysregulation of the neurovascular–immune axis as a unifying pathogenic thread across acute injury and recovery.

Unlike cardiac or hepatic tissues, the adult brain has limited structural and functional regenerative capacity [307, 308]. Once neuronal loss crosses an irreversible threshold, recovery relies largely on network plasticity and compensation rather than cellular replacement. Consequently, glial responses become key determinants of outcome. Persistent microglial proinflammatory activation can drive chronic neurodegeneration through sustained cytokine signaling and synaptic pruning, whereas reactive astrocytic scar formation restricts inflammatory spread but simultaneously imposes a physical and molecular barrier to axonal regeneration and circuit reorganization. These dual roles create a key therapeutic tension between early barrier protection and later modulation of chronic neuroinflammation and glial scarring [309].

Importantly, cerebral IRI is not confined to the central nervous system. BBB disruption and release of brain‐derived DAMPs can remodel systemic immunity through circulating and neurohumoral pathways [310, 311]. Vagal afferent signaling and altered autonomic balance contribute to redistribution of immune cell pools across the spleen, bone marrow, and gut‐associated lymphoid tissues, helping to explain post‐stroke lymphopenia and heightened susceptibility to infection [312, 313]. Conversely, circulating IL‐6, TNF‐α, NET fragments, and complement activation products can injure peripheral organs—including the liver, kidney, and lung—thereby driving bidirectional dysregulation of the brain–heart and brain–gut axes and reinforcing the concept of stroke as a systemic disease [314, 315].

In summary, the essence of cerebral IRI is a temporally and spatially layered disintegration of the neurovascular–immune network. The acute phase is dominated by neuronal injury, BBB failure, and immunothrombosis, whereas the recovery phase is shaped by chronic inflammation and maladaptive neuroglial remodeling; both phases propagate systemic immune remodeling and contribute to distant organ outcomes. Accordingly, future neuroprotective strategies after stroke reperfusion should integrate rapid recanalization with preservation of barrier homeostasis, modulation of NET and immunothrombus burden, and precision management of systemic immune–metabolic states [316, 317]. Such comprehensive approaches are most likely to reduce disability and long‐term multiorgan complications [318].

### 5.4 Renal IRI

Renal IRI is common in kidney transplantation, major cardiac surgery, septic shock, and other intensive care settings [319, 320]. Clinically, it manifests as acute tubular injury and DGF and can accelerate progression to CKD [321, 322]. This vulnerability arises from the convergence of corticomedullary oxygen gradients, energy‐metabolic dependencies, and a highly reactive innate immune microenvironment. The outer medulla, which bears a substantial tubular reabsorptive load, receives relatively low blood flow and operates near the threshold of hypoxia under physiological conditions; it therefore readily crosses into irreversible injury during systemic hypoperfusion or transient redistribution of perfusion.

Proximal tubular epithelial cells are mitochondria‐rich and depend heavily on fatty acid β‐oxidation to sustain active transport. During ischemia, interruption of oxygen delivery and substrate oxidation rapidly depletes ATP, disabling the Na^+^/K^+^‐ATPase and Ca^2^^+^ pumps. This energy failure disrupts cytoskeletal architecture and tight junctions, causes brush‐border loss, and promotes cast formation that mechanically obstructs tubular lumens. Although macroscopic flow is restored during reperfusion, intrarenal perfusion remains highly heterogeneous at the nephron and microvascular levels.

A defining organ‐specific feature of renal IRI is susceptibility to ferroptosis, reflecting distinctive iron handling and lipid composition [323, 324]. Proximal tubular epithelium expresses high levels of transferrin receptor and other iron transporters, and its membrane phospholipids are enriched in polyunsaturated fatty acids—providing abundant substrates for iron‐catalyzed lipid peroxidation [325, 326]. During ischemia, antioxidant defenses collapse: cystine uptake and glutathione synthesis are constrained, and GPX4 activity is reduced, limiting detoxification of lipid hydroperoxides. Upon reperfusion, expansion of the labile iron pool accelerates lipid radical chain reactions, producing characteristic ferroptotic ultrastructural changes in tubules (e.g., condensed mitochondria with increased membrane density). Ferroptotic injury promotes epithelial sloughing and tubular obstruction and releases oxidized lipid‐rich danger signals that activate peritubular immune cells and endothelial cells, thereby converting metabolic injury into amplified inflammation.

Microvascular endothelial injury constitutes a second major vulnerability [327]. Glomerular and peritubular capillary endothelial cells depend on complement‐regulatory mechanisms to maintain immune quiescence. Excess complement activation during reperfusion promotes deposition of terminal membrane attack complexes, inducing endothelial calcium overload, contraction, and detachment. Increased permeability with interstitial edema and erythrocyte extravasation further compresses peritubular capillaries, worsening outer medullary hypoperfusion and producing a “reperfusion without perfusion” no‐reflow phenotype [328].

The medullary microcirculation—characterized by low flow velocity and high viscosity—also favors leukocyte retention and NET formation [329, 330]. Neutrophils activated by renal DAMPs, complement fragments, and platelet‐derived cues release NETs within peritubular capillaries and vasa recta [331, 332]. NET scaffolds mechanically obstruct microvessels and deliver histones and proteases that directly injure endothelium and tubular epithelium, thereby shifting potentially reversible dysfunction toward structural necrosis. NET‐associated nucleic acids and proteases further promote immunothrombosis through contact activation (factor XII) and TF‐dependent pathways, pushing the medullary circulation from hypoperfusion toward occlusion.

Tubular epithelial cells are not passive targets but active immune sensors and effectors [333, 334]. Cytoskeletal disruption, mitochondrial injury, and lysosomal stress upregulate pattern‐recognition receptors (e.g., TLR4) and activate NF‐κB and NLRP3 signaling, promoting production of IL‐1β and IL‐18 [335, 336]. Tubules also shape the local immune milieu through release of injury markers and mediators such as kidney injury molecule‐1 (KIM‐1) and NGAL [337, 338]. When autophagic flux is impaired early in reperfusion—often due to lysosomal dysfunction—damaged mitochondria accumulate, and p62 signaling amplifies oxidative and inflammatory stress. The convergence of multiple RCD programs, including ferroptosis, pyroptosis, and necroptosis, thus positions the tubular epithelium as both an origin of injury and a driver of downstream inflammatory and fibrotic remodeling [339, 340].

Myeloid responses show phase‐dependent effects. Early inflammatory macrophages amplify endothelial injury and coagulation through reactive oxygen species generation and tissue factor expression [341]. Failure to transition toward reparative phenotypes in later phases sustains profibrotic signaling (including TGF‐β pathways), driving fibroblast activation, myofibroblast accumulation, collagen deposition, and capillary rarefaction. This maladaptive repair trajectory provides a mechanistic basis for progression from AKI to CKD [342, 343].

NET burden also exhibits spatial heterogeneity: cortical NETs may primarily reflect systemic inflammation and circulating neutrophil activation, whereas medullary NETs are more tightly linked to local hypoxia, urate crystal deposition, and complement activation [344, 345]. Higher NET deposition associates with slower functional recovery and steeper long‐term decline in glomerular filtration.

Clinically, renal IRI is typically detected by rising serum creatinine, altered urine output, and urinary casts; however, these indices lag behind early molecular injury. Biomarkers such as NGAL, KIM‐1, and IL‐18 can identify tubular injury earlier, but they do not discriminate among RCD programs or quantify immunothrombotic burden, limiting precision deployment of ferroptosis inhibitors, iron chelators, or NET‐directed therapies. Moreover, renal IRI extends beyond the kidney: cytokines, extracellular vesicles, and NET fragments released from injured tubules and endothelium enter the circulation and can promote endothelial activation and immunothrombosis in distant organs, contributing to kidney–heart, kidney–lung, and kidney–liver crosstalk and amplifying MODS [346, 347].

In summary, renal IRI reflects destabilization of a tightly coupled metabolic–immune–microcirculatory triad within a distinctive structural context [348, 349]. Predisposition to ferroptosis, the immune‐sensing capacity of tubular epithelium, and the intrinsic fragility of the medullary microcirculation collectively confer extreme sensitivity to transient ischemia [350, 351]. If early metabolic and microvascular disturbances are not corrected, inflammatory and fibrotic programs rapidly consolidate and propagate systemically. Future renoprotective strategies will likely require coordinated inhibition of ferroptosis and oxidized lipid signaling, reduction of NET‐driven immunothrombosis, and concurrent support for tubular repair and capillary preservation to interrupt progression from AKI to CKD and multiorgan failure [352, 353].

### 5.5 Lung, Gut, Skeletal Muscle, and Systemic IRI

Despite their distinct anatomical locations, the lung, gut, and skeletal muscle share a common pathological theme in IRI, characterized by barrier dysfunction and amplification of systemic inflammation. Their vulnerability is shaped by organ‐specific microenvironment–immune interactions and contributes to the development of MODS through interorgan crosstalk.

Pulmonary IRI frequently arises during lung transplantation, extracorporeal membrane oxygenation weaning, and shock resuscitation [354, 355]. Its pathological core is disruption of the alveolar–capillary barrier. During ischemia, pulmonary microvascular endothelial junctional proteins (e.g., VE‐cadherin) undergo internalization, whereas reperfusion promotes NET deposition within the alveolar space. NET‐derived histone H4 induces GSDMD‐dependent pyroptosis of alveolar epithelial cells, while complement anaphylatoxins C3a and C5a activate alveolar macrophages to release IL‐1β, exacerbating ARDS [356, 357]. Organ‐specific features include pulmonary capillary hypertension, which predisposes to noncardiogenic pulmonary edema, and impaired regenerative capacity of alveolar Type II cells secondary to TGF‐β1 signaling, promoting fibrotic remodeling.

Intestinal IRI, commonly caused by mesenteric artery embolism or systemic shock, poses unique challenges owing to the fragility of the mucosal barrier [358, 359]. Ischemia induces degradation of epithelial tight junction proteins such as occludin, while reperfusion‐associated DAMP release activates intestinal macrophages via TLR9, promoting NET formation and disrupting Paneth cell‐derived antimicrobial peptide secretion [360, 361]. These events facilitate microbial translocation and endotoxemia, triggering systemic inflammatory response syndrome (SIRS). The high metabolic demand of villus tips renders them particularly susceptible to ischemic stress, and mesenteric lymphatic pathways function as conduits for dissemination of inflammatory mediators to distant organs.

Skeletal muscle IRI is most prominent in traumatic crush injuries and following vascular reconstruction. Pathogenesis involves mitochondrial Ca^2^^+^ overload and sarcoplasmic reticulum stress in myocytes, leading to release of myoglobin, potassium ions, and DAMPs into the circulation. These mediators contribute directly to acute tubular necrosis and hyperkalemic arrhythmias [362, 363]. In addition, muscle interstitial edema compresses the microvasculature, producing secondary ischemia, while NETs released by activated neutrophils engage the complement system through MPO–DNA complexes, amplifying injury in remote organs [364, 365].

Systemic crosstalk in IRI is mediated by complex interorgan signaling networks [366]. Deficiency of gut microbiota‐derived metabolites weakens the anti‐inflammatory capacity of hepatic Kupffer cells; pulmonary IL‐6 promotes myelopoiesis, exacerbating renal tubular inflammation; and succinate released during skeletal muscle injury activates hepatic inflammasomes via the circulation [367, 368]. Clinically, plasma cfDNA and CitH3 levels correlate strongly with Sequential Organ Failure Assessment scores in critically ill patients, and elevations in liver enzymes following intestinal ischemia predict adverse outcomes [369, 370]. IRI in these organs thus manifests not only as localized dysfunction but also as dynamic nodes within an integrated immunometabolic network. The strength and pattern of this network are further modulated by host factors such as age and diabetes—for example, diabetes‐associated glycosylation of intestinal tight junction proteins increases barrier permeability, whereas aging livers exhibit reduced clearance of circulating DAMPs.

**FIGURE 3:**
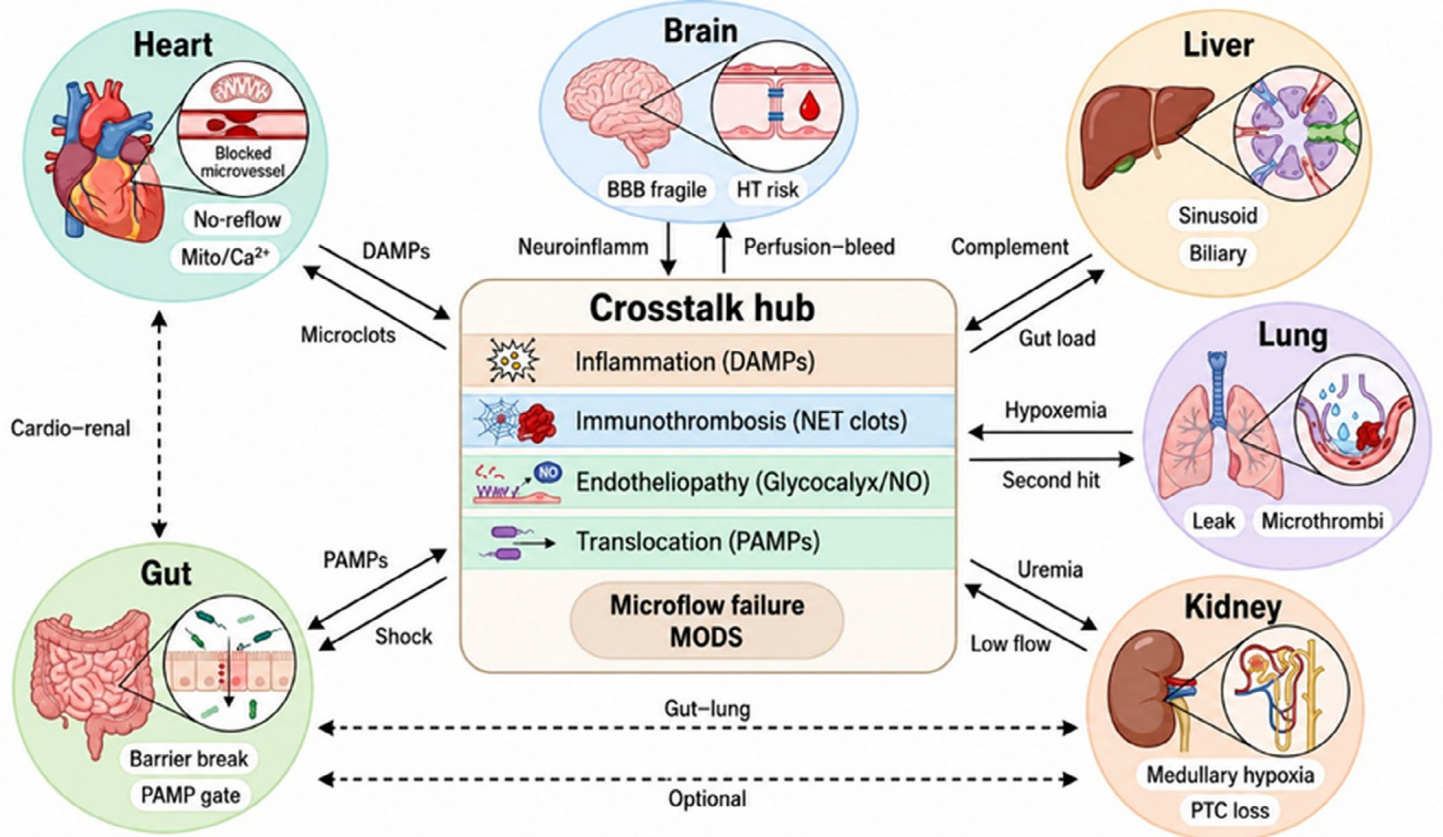
Organ‐specific vulnerable structures and systemic crosstalk pathways shaping multiorgan IRI. Despite shared upstream mechanisms, organ phenotypes of IRI are dictated by vulnerable microanatomical niches, including microvascular beds, barrier interfaces, and resident immune environments. The heart is constrained by coronary microcirculatory “no‐reflow” and cardiomyocyte mitochondrial/Ca^2^^+^ stress, whereas the brain is governed by the neurovascular unit and blood–brain barrier integrity with reperfusion‐associated hemorrhagic risk. The liver centers on sinusoidal endothelium and biliary susceptibility, and the kidney on hypoxia‐prone tubular segments and peritubular capillaries. The lung is defined by the alveolar–capillary barrier and pulmonary microthrombosis, while the gut functions as a key translocation gateway upon barrier collapse. These organ niches are linked by dominant systemic axes—(i) inflammatory spillover, (ii) immunothrombotic dissemination, (iii) endothelial dysfunction‐driven microcirculatory failure, and (iv) barrier breach‐mediated translocation—together propagating remote organ injury and multiorgan dysfunction. BBB, blood–brain barrier; Ca^2+^, calcium ion; DAMPs, damage‐associated molecular patterns; HT, hemorrhagic transformation; IRI, ischemia–reperfusion injury; Mito, mitochondria; MODS, multiple organ dysfunction syndrome; NETs, neutrophil extracellular trap; NO, nitric oxide; PAMPs, pathogen‐associated molecular patterns; PTC, peritubular capillary.

Collectively, these observations demonstrate that single‐organ protective strategies are often insufficient because they fail to account for systemic and distant‐organ effects. Future therapeutic approaches must integrate interorgan communication pathways, particularly along the gut–liver axis and microbiota‐mediated signaling (Figure 3) [371, 372]. This systems‐level perspective provides a pathophysiological framework for integrating preclinical and clinical evidence in subsequent sections and underscores the necessity of mechanism‐driven systemic therapies to overcome current translational barriers (Table 3).

**TABLE 3:** Organ‐specific mechanisms and outcomes of ischemia–reperfusion injury.

Organ	Key mechanism	Vulnerable structures	NET feature	Long‐term outcome	References
Heart	No‐reflow and microthrombosis	Coronary microvasculature	NET–fibrin scaffolds stabilize thrombi; DNase improves perfusion	Adverse remodeling; heart failure risk	[373, 374, 375]
Brain	Postrecanalization immunothrombosis	Distal microvessels; penumbra	NET‐rich thrombi reduce lysis/recanalization efficiency; NET burden tracks severity	Hemorrhagic transformation; worse functional recovery	[376]
Liver	Innate immune amplification after ischemia	Sinusoidal endothelium; cholangiocytes	NETosis couples with platelet/complement loops; NET susceptibility links to graft injury	EAD; biliary injury; chronic graft damage	[377, 378, 379]
Kidney	Outer‐medullary vulnerability; complement/endothelial injury	Proximal tubules; peritubular capillaries	PAD4‐NET axis drives AKI; C3aR promotes NETosis; NET histones worsen tubular necrosis	AKI‐to‐CKD transition; fibrosis; capillary rarefaction	[380, 381, 382]
Lung	Alveolar–capillary barrier failure	Alveolar epithelium; microvascular endothelium	NET accumulation in PGD/LIRI; GSDMD promotes NET formation	PGD/ARDS; profibrotic remodeling	[383, 384, 385]
Gut	Barrier breakdown → systemic inflammation	Villus‐tip epithelium; tight junctions	NETs mediate gut–lung axis injury; oxidative injury amplifies barrier loss	SIRS/MODS; chronic inflammation risk	[386]
Skeletal muscle	Crush/reperfusion inflammatory cascade	Microvasculature; compartment space	NET–TLR signaling; NET clearance improves limb injury	Fibrosis; incomplete functional recovery	[387]

Abbreviations: AKI, acute kidney injury; ARDS, acute respiratory distress syndrome; C3aR, complement component 3a receptor; CKD, chronic kidney disease; DNase, deoxyribonuclease; EAD, early allograft dysfunction; GSDMD, gasdermin D; LIRI, lung ischemia–reperfusion injury; MODS, multiple organ dysfunction syndrome; NETs, neutrophil extracellular traps; PAD4, peptidyl arginine deiminase 4; PGD, primary graft dysfunction; SIRS, systemic inflammatory response syndrome; TLR, Toll‐like receptor.

## 6 Preclinical and Clinical Evidence: Chains and Gaps

This section evaluates the evidence chain connecting mechanistic discovery with clinical translation. It first summarizes experimental models that test causal modules, then reviews representative clinical trials and translational studies targeting mitochondria, complement, NETs, remote ischemic conditioning, and machine perfusion [388, 389]. The goal is to identify which mechanisms are supported by interventional evidence and which remain mainly associative.

### 6.1 Typical Preclinical Animal Models

From the perspective of evidence‐chain construction, the primary value of preclinical models in IRI research lies not merely in “replicating the ischemia–reperfusion process,” but in validating causal relationships between mechanistic modules and identifying translatable therapeutic targets through precise control of ischemia duration, reperfusion kinetics, and tissue microenvironmental variables. Classic multiorgan IRI models are generally structured around three core dimensions: (i) metabolic priming during ischemia and amplification of transient reperfusion injury; (ii) microcirculatory perfusion failure and immune thrombogenesis; and (iii) injury discrimination and long‐term remodeling trajectories.

In myocardial IRI, the coronary artery ligation–reperfusion model is widely used not only to quantify infarct size but also to elucidate how ischemic metabolic substrates shape reperfusion‐associated ROS bursts and determine downstream cell death programs [390, 391]. A representative conceptual framework—originally proposed in a seminal Nature study—describes a conserved cascade of “succinate accumulation during ischemia → Complex I RET upon reperfusion → ROS burst [392, 393].” This mechanism has been validated in both myocardial and cerebral ischemia models, providing a high‐resolution causal chain supporting a cross‐organ paradigm linking metabolism, mitochondrial dysfunction, and inflammatory cell death.

In cerebral IRI research, the transient middle cerebral artery occlusion model offers the advantages of controllable hemodynamics and preserved neurovascular unit architecture, making it particularly suitable for investigating blood–brain barrier disruption, immune thrombosis, and NET‐mediated HAT. Recent animal studies further indicate that inhibition of PAD4 confers neuroprotection, elevating NETs from a purely correlative marker to a candidate interventional mechanistic module [394, 395].

Renal IRI models, most commonly established via renal artery clamping, enable integrated assessment of proximal tubular metabolic vulnerability, microcirculatory perfusion heterogeneity, and the continuum from AKI to CKD within a single experimental system [396, 397]. Notably, genetic and pharmacological evidence supporting a causal role for NETs is particularly strong in renal IRI. For example, PAD4 deficiency or DNase I‐mediated NET degradation consistently improves renal function and attenuates tissue injury, providing direct evidence for the immune thrombosis–microcirculatory failure axis [398, 399].

Hepatic IRI is typically modeled using portal vein occlusion or partial hepatic ischemia. These systems uniquely integrate sinusoidal endothelial injury, rapid Kupffer cell‐mediated inflammatory responses, and complement–coagulation interactions, making them well suited for dissecting how immune thrombosis and perfusion failure synergistically disrupt sinusoidal architecture. In parallel, transplantation‐associated IRI has driven the development of machine perfusion platform models, extending experimental interventions from in vivo drug administration to ex vivo perfusion‐based delivery [400, 401]. These platforms offer highly controllable settings for evaluating complement inhibition, anti‐inflammatory therapies, and immune‐thrombosis‐targeted strategies.

Pulmonary IRI models predominantly emphasize endothelial glycocalyx degradation, increased microvascular permeability, and neutrophil extravasation, illustrating how injury to barrier organs can rapidly propagate into systemic inflammation. In contrast, gut and skeletal muscle IRI models are frequently employed to study the amplification axis linking local reperfusion injury to systemic inflammatory responses and distant organ dysfunction, thereby providing mechanistic insight into the development of MODS in clinical contexts such as trauma resuscitation and limb reperfusion.

Collectively, these models underscore that preclinical studies with high translational potential should move beyond descriptive replication of pathology. Instead, they should be designed to construct testable causal chains centered on mechanistic modules, incorporating multiorgan comparisons, genetic manipulation, and quantitative microcirculatory phenotyping to enhance interpretability and predictive power along the pathway from mechanism to clinical application.

### 6.2 Clinical Trials and Representative Studies

At the clinical research level, therapeutic development for IRI has long been constrained by the well‐recognized dilemma of “strong mechanisms but weak translation.” Extensive preclinical evidence implicates mitochondrial dysfunction, oxidative stress, and immunothrombosis as central pathogenic modules. However, in real‐world clinical trials, single‐target interventions have often failed to reproducibly achieve the protective effects observed in animal models. This translational gap likely reflects multiple factors, including narrow therapeutic time windows, marked patient heterogeneity, suboptimal outcome selection, and redundancy among interacting injury pathways.

Myocardial IRI provides a representative example. Clinical strategies targeting mPTP opening—most notably peri‐reperfusion administration of cyclosporine A in ST‐segment elevation myocardial infarction (STEMI)—initially showed promise in reducing infarct size [402, 403]. Nevertheless, subsequent large randomized trials, such as CIRCUS, failed to demonstrate consistent benefits on hard clinical endpoints. These largely negative findings prompted critical reassessment, underscoring that the efficacy of mitochondrial‐targeted therapies depends on variables such as timing of reperfusion, severity of microvascular obstruction, and underlying immunothrombotic burden. Restoration of epicardial blood flow alone does not equate to effective inhibition of downstream injury mechanisms [404].

In ischemic stroke, the advent of mechanical thrombectomy has shifted the focus of IRI research from simple vessel recanalization to postreperfusion injury and the immunothrombotic architecture of occlusive thrombi [405, 406]. Analyses of retrieved thrombi consistently demonstrate enrichment of NETs, suggesting that NETs enhance thrombus stability and resistance to fibrinolysis, thereby impairing reperfusion quality and functional recovery [407, 408]. In parallel, circulating NET‐associated biomarkers, such as MPO–DNA complexes, correlate with adverse outcomes, offering measurable indicators of an “immunothrombotic stroke” phenotype [409, 410].

Clinical investigation of transplant‐associated IRI has increasingly focused on complement activation and perfusion strategies. Systematic reviews support a pathogenic role for complement in transplant IRI, and complement‐targeting agents—already in clinical use for several complement‐mediated disorders—provide a practical pharmacological basis for extension into this context. Moreover, advances in machine perfusion technology have created platforms for ex vivo delivery and real‐time assessment of interventions, enabling trial designs to move beyond static cold preservation toward more mechanism‐focused evaluation [411, 412]. In kidney transplantation and AKI, clinical studies demonstrate associations among complement activation, inflammatory burden, and DGF [413, 414]. Nonetheless, consensus regarding effective complement‐directed therapies remains elusive, highlighting persistent gaps between robust mechanistic insights and incomplete clinical evidence chains.

In parallel, clinical imaging studies emphasizing immunothrombosis and microcirculatory phenotypes are reshaping outcome assessment in IRI. In myocardial infarction, for example, microvascular obstruction and intramyocardial hemorrhage are now recognized as critical intermediate phenotypes closely linked to long‐term heart failure risk [415, 416]. These microcirculatory endpoints offer a more mechanistically informative bridge between experimental findings and clinical outcomes than infarct size alone [417, 418]. Collectively, current evidence suggests that successful translational strategies in IRI will require mechanism‐oriented patient stratification and composite endpoint design, explicitly incorporating immunothrombotic and microcirculatory phenotypes rather than relying solely on reperfusion status or conventional organ function metrics.

Representative registered trials and translational studies that support this clinical discussion are summarized in Table 5.

### 6.3 Integration of the Mechanism–Intervention–Evidence Chain and Research Gaps

Integration of preclinical and clinical data reveals that the principal limitation in IRI research is not a lack of therapeutic targets but the absence of a coherent, mechanism‐aligned evidence loop. Within the metabolic–mitochondrial module, ischemia‐induced succinate accumulation and subsequent RET‐driven ROS generation upon reperfusion constitute a highly conserved, cross‐organ pathogenic cascade [419]. Animal studies demonstrate that modulating succinate metabolism or inhibiting RET can attenuate tissue injury. Clinically, however, the absence of real‐time biomarkers reflecting module activity and the inability to precisely control therapeutic timing have hindered consistent efficacy of mitochondrial‐targeted interventions.

Similarly, in the immunothrombotic module, convergent evidence from experimental models and human samples supports positive feedback interactions among NETs, complement, and platelets. Yet, key gaps persist: NET‐associated biomarkers lack specificity, and most clinical studies remain correlational. Mechanism‐driven trials that prospectively enroll patients based on high immunothrombotic or NET‐burden phenotypes are notably lacking, limiting causal validation. In transplant‐associated IRI, complement inhibitors offer feasible therapeutic tools, and machine perfusion platforms enable targeted delivery and mechanistic interrogation [420, 421]. However, heterogeneity related to organ type, donor characteristics, and preservation strategies complicates interpretation of trial outcomes. Integration of complement activation profiles with microcirculatory perfusion phenotypes into a unified evaluative framework is urgently needed.

Accordingly, the most pressing unmet need is not the identification of additional intervention targets, but the establishment of cross‐organ, comparable mechanistic phenotypes; alignment of time‐phased interventions with appropriate endpoints; and systematic clarification—through structured evidence synthesis—of which mechanistic modules are supported by strong causal data and which remain at the level of suggestive association.

Building on this foundation, future IRI research is likely to evolve from “one‐size‐fits‐all” monotherapies toward a strategic paradigm integrating mechanistic modules, phenotypic stratification, and rational combination therapies. Such an approach holds the greatest promise for improving the predictability, reproducibility, and clinical impact of translational IRI research (Table 4).

**TABLE 4:** Mechanistic modules, intervention strategies, and preclinical and clinical evidence in multiorgan ischemia–reperfusion injury.

Mechanistic module	Intervention strategy	Preclinical evidence	Clinical evidence	Conclusions and limitations	References
Mitochondrial metabolic dysfunction and reperfusion oxidative stress	Inhibition of succinate accumulation and reverse electron transport	Across multiple organs, ischemic succinate accumulation drives mitochondrial reactive oxygen species generation during reperfusion. Metabolic modulation markedly reduces tissue injury in experimental models.	Mitochondria‐targeted metabolic interventions remain largely in early translational stages with limited clinical validation.	The mechanism is highly conserved, but lack of real‐time metabolic phenotyping and precise timing control limits clinical translation.	[422, 423]
Mitochondrial permeability transition pore opening	Inhibition of permeability transition pore opening	Experimental studies demonstrate reduced infarct size and attenuated reperfusion injury with permeability transition pore inhibition.	Large clinical trials in myocardial infarction have failed to show consistent improvement in hard clinical endpoints.	Population heterogeneity and concurrent therapies likely diminish the effectiveness of single‐target mitochondrial interventions.	[424, 425, 426]
Endothelial glycocalyx degradation and barrier dysfunction	Protection and restoration of the endothelial glycocalyx	Reperfusion induces glycocalyx shedding, leading to microcirculatory impairment. Preserving glycocalyx integrity improves endothelial function in experimental models.	Clinical evidence is mainly associative and derived from transplantation and critical care cohorts.	Standardized glycocalyx biomarkers and prospective interventional trials are currently lacking.	[427, 428]
Excessive complement activation	Inhibition of complement initiation or terminal pathways	Complement inhibition reduces endothelial injury and inflammatory amplification in diverse ischemia–reperfusion models.	Kidney transplantation studies indicate feasibility, but randomized trials show inconsistent efficacy.	Therapeutic benefit must be balanced against infection risk and cost, and may require complement phenotype stratification.	[429, 430, 431]
Neutrophil extracellular trap driven immunothrombosis	Degradation of extracellular traps or inhibition of their formation	Removal or suppression of extracellular traps improves microvascular perfusion and limits tissue damage in multiple organ models.	Prospective interventional trials enrolling patients based on immunothrombotic phenotypes are currently lacking.	Limited biomarker specificity, narrow therapeutic windows, and unresolved safety concerns constrain translation.	[432, 433, 434]
Sustained inflammatory amplification	Precision anti‐inflammatory strategies	Experimental and human studies support a central role of inflammatory amplification in driving tissue injury and disease progression.	Anti‐inflammatory therapies reduce cardiovascular events in selected populations, but ischemia–reperfusion‐specific trials are scarce.	Anti‐inflammatory approaches require closer alignment with ischemia–reperfusion‐specific mechanisms and temporal dynamics.	[435, 436]

Table 5 extends this mechanism‐based framework by mapping representative clinical trials and translational studies to the intervention modules discussed above.

**TABLE 5:** Representative clinical trials and translational studies targeting ischemia–reperfusion injury.

Strategy and clinical context	NCT no./identifier; status/phase	Objective	Preliminary findings or available outcomes	References
Cyclosporine A for mitochondrial permeability transition pore inhibition in ST‐segment elevation myocardial infarction treated with primary percutaneous coronary intervention	NCT01502774 (CIRCUS); completed; Phase 3	Test whether cyclosporine before reperfusion improves clinical outcomes after acute myocardial infarction.	Large randomized evidence did not show consistent improvement in hard clinical endpoints, illustrating the limits of isolated mitochondrial targeting without phenotype stratification.	[437, 438, 439]
Cyclosporine A for mitochondrial permeability transition pore inhibition in reperfused acute myocardial infarction	NCT01650662 (CYCLE); completed; Phase 3	Evaluate whether a single intravenous bolus before percutaneous coronary intervention improves myocardial reperfusion and injury markers.	Results across cyclosporine studies were inconsistent, reinforcing the importance of timing, microvascular patency, and patient heterogeneity.	[440, 441, 442]
Dornase alfa/deoxyribonuclease for neutrophil extracellular trap degradation in large‐vessel ischemic stroke treated with thrombolysis and endovascular thrombectomy	NCT05203224 (EXTEND‐IA DNase); recruiting; Phase 2	Dose‐finding trial testing whether intravenous dornase alfa improves early angiographic reperfusion before thrombectomy.	No posted results at the time of revision; directly evaluates neutrophil extracellular trap degradation as an adjunct reperfusion strategy.	[443, 444, 445]
Remote ischemic conditioning in acute ischemic stroke treated with mechanical thrombectomy	NCT06559241 (RECAST‐MT); recruiting; Phase 3	Evaluate the safety and efficacy of remote ischemic conditioning and explore treatment duration after thrombectomy.	No posted results at the time of revision; tests noninvasive reperfusion modulation and systemic organ‐protective signaling.	[446, 447, 448]
Normothermic machine perfusion with viability assessment for initially declined donor liver grafts	NCT06874296 (ExTra); recruiting; not applicable	Assess whether normothermic machine perfusion safely increases use of declined livers and reduces waiting time.	No posted results at the time of revision; provides an ex vivo platform for organ assessment and targeted intervention before transplantation.	[449, 450, 451]
Hypothermic oxygenated perfusion for extended‐criteria donor liver transplantation	NCT03929523 (HOPExt); completed; not applicable; results submitted	Evaluate hypothermic oxygenated perfusion in grafts vulnerable to early allograft dysfunction and primary nonfunction.	Relevant to reperfusion regulation, oxygen delivery, and biliary or graft‐injury endpoints in liver transplantation.	[452, 453, 454]
Eculizumab for terminal complement blockade in delayed graft function after deceased‐donor kidney transplantation	NCT02145182 (PROTECT); completed; Phase 2/3	Test whether C5 blockade prevents delayed graft function in adults at increased risk.	Completed without consistent clinical efficacy; supports complement‐activation phenotyping rather than empiric broad enrollment.	[455, 456, 457]
Peri‐transplant eculizumab for complement blockade in deceased‐donor kidney transplantation at risk for delayed graft function	NCT01919346; terminated; Phase 2	Evaluate anti‐C5 therapy administered around transplantation to prevent delayed graft function.	Related pilot trials did not prevent delayed graft function, highlighting redundancy among complement, endothelial injury, and immunothrombosis pathways.	[458, 459, 460]
Oxygenated hypothermic machine perfusion in donation‐after‐circulatory‐death kidney transplantation	ISRCTN32967929 (COMPARE); completed; Phase 3 paired randomized trial	Compare oxygenated with nonoxygenated hypothermic machine perfusion.	The primary 12‐month estimated glomerular filtration rate endpoint was not significantly improved, but severe complications and graft failure were lower in the oxygenated arm.	[461, 462, 463]

Abbreviations: ISRCTN, International Standard Randomised Controlled Trial Number; NCT, National Clinical Trial.

Together, these studies show that interventions directed at a single mitochondrial or complement node have produced inconsistent clinical benefits, whereas platform‐based reperfusion control and NET/immunothrombosis‐directed approaches are increasingly being evaluated with mechanism‐aligned endpoints.

## 7 Mechanism‐Guided Therapeutic Overview

This section translates the preceding mechanisms into therapeutic principles. Rather than treating reperfusion as a binary event, it considers reperfusion as a time‐sensitive window in which hemodynamics, metabolism, endothelial integrity, inflammation, and coagulation can be jointly modulated. Emphasis is placed on biomarker‐guided selection and combination strategies [464].

### 7.1 Reperfusion Regulation and Fundamental Principles of Organ Protection

Optimization of reperfusion strategies constitutes the cornerstone of IRI management. The main objective is not merely restoration of blood flow, but precise regulation of the spatiotemporal dynamics of reperfusion. In clinical practice, controlled or progressive reperfusion approaches—such as postdilatation pressure modulation during coronary interventions—reduce myocardial mPTP opening by avoiding abrupt oxygen surges and excessive mechanical stress [465, 466]. In organ transplantation, machine perfusion technologies enable dynamic control of oxygen tension, substrate delivery, and perfusion pressure waveforms, facilitating clearance of accumulated DAMPs and preservation of endothelial glycocalyx integrity [467, 468]. Notably, the PROTECT trial in liver transplantation demonstrated that normothermic machine perfusion increased the utilization of marginal donor livers by approximately 40% [469, 470].

Organ‐specific protection principles are critical. Myocardial protection prioritizes prevention of calcium overload and maintenance of energy metabolism; cerebral protection requires tight regulation of blood pressure to preserve cerebral perfusion pressure while avoiding hyperoxia‐induced ROS bursts; hepatic protection focuses on maintaining an optimal hepatic artery–portal vein flow balance and minimizing biliary ischemia; and renal protection emphasizes optimization of electrolyte composition within perfusion solutions [471]. Although ischemic adaptation strategies (e.g., ischemic preconditioning) represent shared protective principles, their efficacy is often attenuated in elderly or diabetic patients, underscoring the need for individualized approaches.

Modification of reperfusion fluid composition—such as supplementation with NO donors to improve microcirculatory flow or SOD mimetics to scavenge ROS—has demonstrated efficacy in experimental models, although clinical translation must address delivery efficiency and safety. A major translational challenge remains the narrow therapeutic time window: optimal interventions must be initiated at the onset of reperfusion, yet logistical delays in emergency and surgical settings frequently preclude timely application. Accumulating evidence supports multimodal protective strategies. For example, in liver transplantation, combining machine perfusion with targeted pharmacological interventions (e.g., complement inhibitors) yields superior protection compared with single‐modality approaches [472, 473]. Collectively, these observations indicate that effective organ protection requires integration of hemodynamic optimization, metabolic support, and immune modulation. They further highlight the importance of precise phenotypic stratification, as intensified anti‐inflammatory strategies are likely to benefit only selected patients with defined immunothrombotic profiles.

### 7.2 Mechanism‐Driven Intervention Strategies

Mechanism‐driven interventions for IRI can be conceptualized as targeted disruption of key nodes within the interconnected cascade linking metabolic and mitochondrial dysfunction, RCD, sterile inflammation, and immunothrombosis. The goal is not simply suppression of inflammation or scavenging of ROS, but reduction of the threshold for irreversible injury during early reperfusion and interruption of self‐amplifying feedback loops.

In myocardial IRI, inhibition of the mPTP has long been regarded as a means to prevent mitochondrial collapse [474, 475]. Agents such as cyclosporine A showed infarct size reduction in animal studies and early‐phase clinical trials; however, larger randomized studies failed to demonstrate consistent benefits on hard clinical endpoints [476, 477]. These results underscore the strong dependence of mitochondrial‐targeted therapies on therapeutic timing, microcirculatory patency, and patient heterogeneity, highlighting the limitations of single‐target, single‐endpoint strategies in the context of the complex IRI network.

In contrast, strategies targeting NETs and complement activation align more closely with the modular pathophysiology of intravascular inflammation and immune thrombosis. Preclinical studies demonstrate that DNase‐mediated NET degradation reduces microvascular obstruction, improves tissue perfusion, and synergizes with thrombolytic therapies [478]. Emerging approaches employing targeted DNase delivery—such as in intestinal IRI models—provide a translatable framework for structural dismantling of immune‐thrombotic scaffolds [479, 480]. Complement inhibition represents another promising strategy: extensive evidence supports its upstream amplification role in transplant‐associated IRI, endothelial injury, and immune remodeling. Ex vivo machine perfusion offers a defined intervention window, rendering C3, C5, and downstream complement effectors pharmacologically accessible targets [481, 482]. Although clinical data remain limited, complement blockade uniquely addresses the inflammation–coagulation interface.

Ferroptosis inhibition, targeting lipid peroxidation and GPX4‐dependent systems, is conceptually aligned with the emerging “metabolism–oxidative injury–membrane disruption” framework of RCD [483, 484]. Sustained interest across renal, cardiac, and hepatic IRI models suggests that ferroptosis modulation may indirectly attenuate inflammatory amplification by limiting lipid oxidation cascades [485]. Modulation of autophagy and mitophagy offers an additional strategy to reduce sterile inflammation at its source by improving mitochondrial quality control and limiting ROS and mitochondrial DNA release; however, these interventions exhibit strong phase dependence and must be applied within well‐defined mechanistic windows.

Overall, the prevailing trend in IRI therapy is shifting from isolated single‐target inhibition toward rational modular combination strategies [486, 487]. Examples include coupling mitochondrial protection with immune‐thrombotic inhibition, integrating complement blockade with microcirculatory support, and combining modulation of cell death pathways with promotion of inflammatory resolution. Correspondingly, outcome evaluation increasingly emphasizes intermediate phenotypes—such as microcirculatory perfusion and immunothrombotic signatures—to improve mechanistic alignment and enhance clinical interpretability.

### 7.3 NET‐ and Immunothrombosis‐Targeted Strategies: Time Window and Safety

Therapeutic strategies targeting NETs and immunothrombosis represent a conceptual shift in IRI research, moving from a predominantly “cell‐autonomous injury” paradigm toward an integrated framework centered on intravascular inflammation–coagulation coupling. However, the clinical feasibility of these approaches is critically dependent on precise delineation of therapeutic time windows and careful evaluation of safety constraints.

NETs form rapidly during the early phase of reperfusion and play a central role in the no‐reflow phenomenon and microcirculatory obstruction. Accordingly, the theoretically optimal intervention window lies between reperfusion onset and stabilization of NET structures within the microvasculature, which may explain why DNase treatment or PAD4 inhibition—both of which suppress NET formation—demonstrate protective efficacy in preclinical models [488, 489]. At the same time, NETs constitute an essential component of innate host defense, and excessive or prolonged suppression may increase susceptibility to infection. This risk is particularly relevant in transplant recipients, critically ill patients, and immunocompromised populations, necessitating cautious risk–benefit assessment.

Immunothrombotic interventions must also navigate the delicate balance between physiological hemostasis and pathological thrombosis [490]. While anticoagulant or antiplatelet therapies may alleviate microvascular obstruction, they concurrently elevate bleeding risk. This safety margin is especially narrow in reperfusion scenarios associated with HAT, such as acute ischemic stroke [491, 492]. Consequently, future strategies are likely to favor localized or organ‐directed approaches—such as ex vivo delivery during machine perfusion or targeted nanocarrier systems—or structural degradation strategies, including DNase‐mediated dismantling of NET scaffolds, to reduce reliance on systemic anticoagulation [493, 494].

Importantly, the success of NET‐ and immunothrombosis‐targeted therapies is closely linked to mechanism‐based patient stratification. Enriching clinical trials with individuals exhibiting a high immunothrombotic burden—identified through NET‐associated biomarkers, microcirculatory obstruction phenotypes, or complement activation signatures—can enhance effect detectability and mitigate dilution of therapeutic efficacy in heterogeneous populations [235, 236]. Overall, progress in this field is unlikely to result from indiscriminate intensification of anticoagulation. Instead, it will depend on the integration of mechanism‐informed biomarkers to guide intervention timing, dosing, and delivery. Such precision approaches aim to attenuate postreperfusion vascular inflammatory amplification and microcirculatory failure while preserving essential host defense and hemostatic functions.

## 8 Conclusion and Future Perspectives

This final section synthesizes the cross‐organ framework and outlines future research priorities. It emphasizes systems‐level immune remodeling, NET‐centered immunothrombosis, biomarker‐stratified clinical trials, and humanized translational platforms such as machine perfusion, organoids, and multimodal data integration.

Research on IRI is evolving from static, single‐organ descriptions toward a cross‐organ network pathophysiology framework. Accumulating evidence indicates that IRI follows a hierarchical cascade, progressing from metabolic derangements, mitochondrial dysfunction, and RCD during ischemia to sterile inflammation and immunothrombosis upon reperfusion. Within this cascade, NET–driven immunothrombosis has emerged as a major hub linking multiorgan injury across the heart, brain, liver, and kidneys.

Despite pronounced organ‐specific manifestations, several core mechanistic modules—including oxidative stress, inflammatory amplification, and microcirculatory dysfunction—exhibit substantial cross‐organ conservation. The principal challenge now lies in translating these mechanistic insights into effective therapies. The repeated failure of ostensibly promising targeted interventions in clinical trials, despite robust preclinical efficacy, underscores the limitations of traditional single‐target, one‐size‐fits‐all strategies and highlights the urgent need for more integrative and precision‐oriented approaches.

Future research should therefore adopt a holistic perspective centered on cross‐organ mechanisms and systemic immune remodeling. IRI is not merely a localized injury but a systemic immune perturbation. Injured tissues release DAMPs, inflammatory mediators, and extracellular vesicles that function as immune messengers, disseminating through the circulation to influence distant organs. These signals reshape immune homeostasis in remote tissues, increasing susceptibility to secondary injury and infection. This phenomenon of cross‐organ immune imprinting provides a mechanistic explanation for clinically observed postreperfusion remote organ dysfunction and long‐term complications such as fibrosis. Accordingly, viewing IRI as a systemic process yields broader insights into disease progression and therapeutic intervention.

Within this framework, the NET–immunothrombotic axis represents a particularly promising cross‐organ therapeutic target. The reticular scaffolds formed by NETs convert localized tissue injury into intravascular inflammation and microcirculatory obstruction, with pathological effects extending to distant organs. Importantly, NET‐associated biomarkers are readily detectable in the circulation and often peak during early reperfusion, aligning closely with clinically actionable intervention windows. Moreover, NET‐mediated injury displays marked organ specificity: for example, pericholedochal NET accumulation in liver transplantation contributes to biliary injury and may be attenuated by DNase supplementation during donor liver machine perfusion, whereas local DNase administration during myocardial IRI can dissolve coronary NETs and improve microvascular perfusion. These observations emphasize that effective NET‐targeted therapies must be tailored to injury timing and organ‐specific microenvironments.

Given the marked heterogeneity of IRI, future clinical trials should prioritize biomarker‐driven precision enrollment, stratifying patients according to dominant pathological mechanisms. For instance, selection for DNase or complement inhibitor therapy could be guided by immunothrombotic biomarker profiles. In parallel, integration of cross‐modal big data and artificial intelligence‐based analytics offers the opportunity to construct predictive models of IRI risk and therapeutic response, thereby supporting individualized clinical decision‐making.

Emerging technological platforms are further accelerating the translation of mechanistic insights into clinical application. Ex vivo machine perfusion enables pretransplant testing and optimization of interventions in isolated organs, providing a unique window for mechanistic validation prior to patient exposure. Human organoids and multiorgan‐on‐chip systems recapitulate key aspects of human organ microenvironments, addressing limitations of animal models and facilitating novel target discovery and high‐throughput drug screening. Together, these technologies are strengthening the translational bridge from bench to bedside.

In summary, IRI research is entering a new phase defined by systems‐level integration. By embracing a cross‐organ perspective, focusing on pivotal pathological axes such as immunothrombosis, implementing biomarker‐stratified precision trial designs, and leveraging advanced platforms including machine perfusion, organoids, and multimodal data integration, the field is poised to overcome longstanding translational barriers. The overarching goal is to transform reperfusion from a trigger of injury into an opportunity for tissue repair. Achieving this vision will require multidisciplinary collaboration and closed‐loop bench‐to‐bedside research. As immunothrombotic phenotypes become increasingly well defined and therapeutically tractable, IRI management can transition from empiricism to personalized organ protection, ultimately improving outcomes for patients with myocardial infarction, stroke, and organ transplantation. Together, these advances may support a transition from empiric reperfusion management to mechanism‐guided organ protection in myocardial infarction, stroke, transplantation, and critical illness.

## Author Contributions

Peng An: writing – original draft, visualization, and validation. Yi An: writing – review and editing and visualization. Mengwei Chen: writing – review and editing and validation. Longlong Wu: conceptualization, funding acquisition, and supervision. Rong Wang: conceptualization, funding acquisition, and supervision. All authors have read and approved the final manuscript.

## Funding

This work was supported by the National Natural Science Foundation of China (82300750) and Shanxi Provincial Natural Science Foundation (202203021221260).

## Ethics Statement

The authors have nothing to report.

## Conflicts of Interest

The authors declare no conflicts of interest.

## Data Availability

Data sharing is not applicable to this article as no datasets were generated or analyzed during the current study.
